# The Influence of Solvent Selection upon the Crystallizability and Nucleation Kinetics of Tolfenamic Acid Form II

**DOI:** 10.1021/acs.cgd.3c00450

**Published:** 2023-07-17

**Authors:** Yu Liu, Cai Y. Ma, Junbo Gong, Kevin J. Roberts

**Affiliations:** † State Key Laboratory of Chemical Engineering, Tianjin University, Tianjin, 300072, China; ‡ Centre for the Digital Design of Drug Products, School of Chemical and Process Engineering, University of Leeds, Woodhouse Lane, Leeds LS2 9JT, U.K.

## Abstract

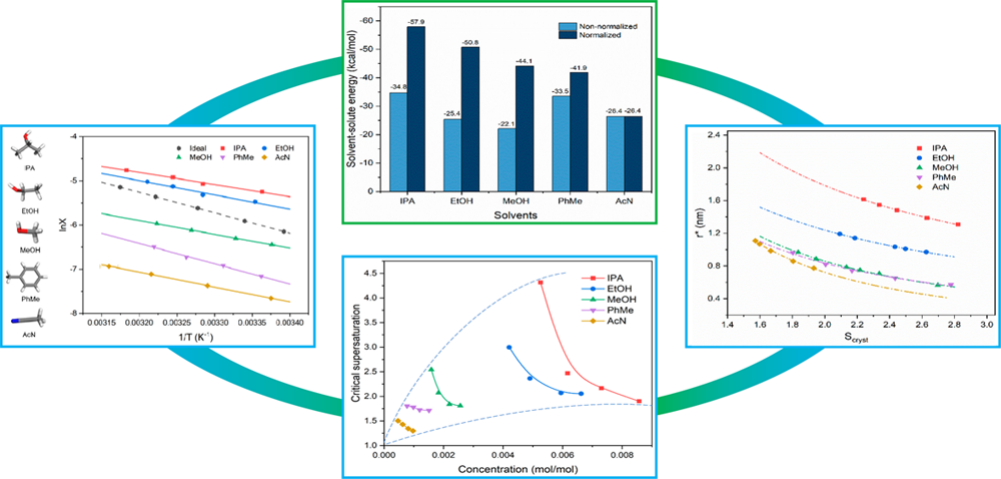

The influence of the solution environment on the solution thermodynamics, crystallizability, and nucleation of tolfenamic acid (TFA) in five different solvents (isopropanol, ethanol, methanol, toluene, and acetonitrile) is examined using an integrated workflow encompassing both experimental studies and intermolecular modeling. The solubility of TFA in isopropanol is found to be the highest, consistent with the strongest solvent–solute interactions, and a concomitantly higher than ideal solubility. The crystallizability is found to be highly dependent on the solvent type with the overall order being isopropanol < ethanol < methanol < toluene < acetonitrile with the widest solution metastable zone width in isopropanol (24.49 to 47.41 °C) and the narrowest in acetonitrile (8.23 to 16.17 °C). Nucleation is found to occur via progressive mechanism in all the solvents studied. The calculated nucleation parameters reveal a considerably higher interfacial tension and larger critical nucleus radius in the isopropanol solutions, indicating the higher energy barrier hindering nucleation and hence lowering the nucleation rate. This is supported by diffusion coefficient measurements which are lowest in isopropanol, highlighting the lower molecular diffusion in the bulk of solution compared to the other solutions. The TFA concentration and critical supersaturation at the crystallization onset is found to be directly correlated with TFA/isopropanol solutions having the highest values of solubility and critical supersaturation. Intermolecular modeling of solute–solvent interactions supports the experimental observations of the solubility and crystallizability, highlighting the importance of understanding solvent selection and solution state structure at the molecular level in directing the solubility, solute mass transfer, crystallizability, and nucleation kinetics.

## Abstract 2

A self-consisted analysis and associated workflow for the crystallization process of TFA cross-correlates solution crystallizability with cooling rate, solution concentration, and solvent type, together with its underpinning molecular scale for the impact of desolvation on nucleation.

## 1 Introduction

Solution crystallization is an effective unit operation for the isolation or purification of solid products and one that has been widely used in the pharmaceutical, chemical, and food industries.^1^ As the first step of crystallization, nucleation is considered to have significant effects on the physical and chemical properties of the final solid products, notably particle size, polymorphic form, and crystallographic perfection.^2^ Therefore, being able to understand and control the nucleation process from the solution phase by solvent selection is of significant current interest.^3,4^

It is known that the solution environment can strongly influence solute mass transfer and the structural nature of the solution (solvation, solute-assembly, and molecular conformations), and can directly impact the overall nucleation process.^5−7^ Hence, probing the relationship between solution state through solvent selection and the nucleation process provides an opportunity to gain a deeper understanding not only of the solute crystallizability but also regarding the mechanism of nucleation from solution. Davey and co-workers^3,8^ proposed the link between the kinetics of nucleation process and desolvation, highlighting that strong solvation strength can decrease the nucleation rate. Research on *p*-aminobenzoic acid, salicylic acid, and risperidone crystallization has also suggested that solute desolvation represents an important rate-limiting step to nucleation.^8−10^ Muller et al.^11^ developed a practical approach for the rapid design of cooling crystallizations based upon the temperature dependence of solubility within over 100 solute–solvent systems. Other studies have highlighted the importance of both molecular and solid-state structures such as aromatic intermolecular stacking and conformation transformation in potentially playing an important role in mediating the nucleation kinetics of some compounds.^12−15^

Nucleation kinetics can be derived through isothermal crystallization studies from the distribution of induction times based on the stochastic nature of nucleation and this has been successfully applied to a number of organic crystals.^12,16,17^ Measurement of metastable zone widths (MSZWs) using polythermal analysis can also provide an effective way to study a compound’s crystallizability and, through this, characterize the effect of the solvent selection process on the kinetics of nucleation and the associated mechanisms.^5,6,18−20^ Knowledge of how MSZWs vary with process scale, crystallizer configuration and crystallization operating conditions can also form important aspects in the design and operation downstream for industrial production.^18^ MSZWs can be characterized through repetitive solution heating–cooling cycles as a function of cooling and heating rates.^5,21−25^ By relating the solution undercooling temperature difference (Δ*T*_*c*_) at the crystallization on-set as a function of the solution cooling rate, the mechanism of nucleation, and the associated kinetics can be derived and analyzed.^26−28^

**Figure 1:**
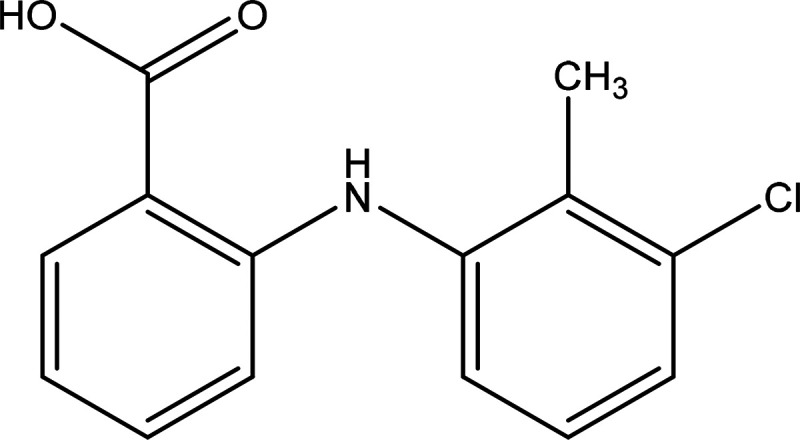
Schematic representation of the molecular structure of tolfenamic acid.

Tolfenamic acid (TFA, Figure 1) is an anti-inflammatory drug with nine reported conformational polymorphs.^29−31^ The most encountered polymorphs are forms I and II, while the other forms have mostly been obtained by using polymer templates.^29^ TFA molecules exhibit similar crystal chemistry and intermolecular packing (carboxylic dimers and infinite aromatic stacking) within its different polymorphic forms, with the main differences between forms I and II being related to the TFA molecule’s different conformations and hence intermolecular packing. As such, TFA provides an important organic model compound for investigating solvent selection, solution structure, and crystallization behavior, in particular their inter-relationship with polymorphic behavior.^32−37^

In this paper, the crystallizability and nucleation kinetics of TFA in five solvents (isopropanol, ethanol, methanol, toluene, and acetonitrile) with different solution properties (apolar, polar protic, and polar aprotic) were investigated using polythermal crystallization analysis.^21−24,38,39^ The data was coupled with solution-state diffusivity measurements as well as intermolecular atom specific grid-search modeling^18,40−42^ of solvation energies and structure. The overall aim of the work has been to examine the influence of the solution state on the crystallization behaviors of TFA and, potentially, explore how the nature of the solution state might, through solvent selection, be able to direct the nucleation and hence the crystallizability of TFA.

## 2 Materials and Methods

**Figure 2:**
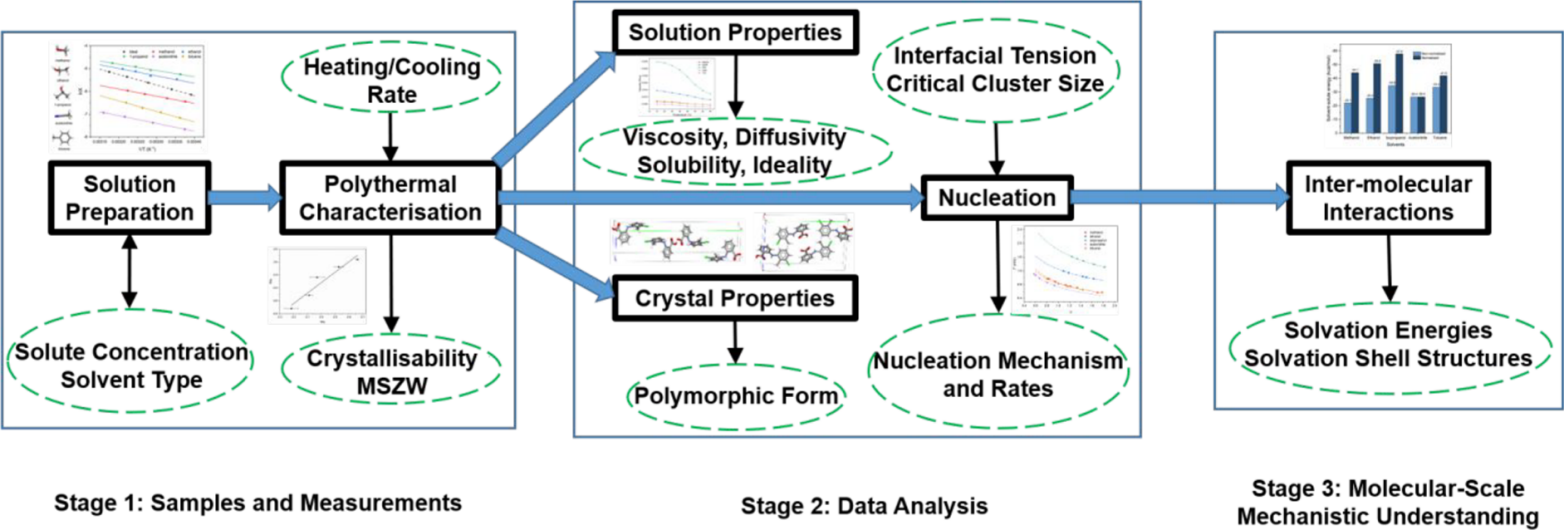
Schematic workflow structure highlighting the interconnectivity between solution and solid-state properties with crystallizability and nucleation highlighting the importance of molecular-scale understanding.

An overview workflow of the methodology is summarized in Figure 2.

### 2.1 Materials

Tolfenamic acid (>99%), form I, was obtained from Fluorochem Ltd. All solvents were purchased from ThermoFisher and of analytical grade. All chemicals were used without further purification.

### 2.2 Experimental Methods

#### 2.2.1 Solution Preparation and Polythermal Experiments

Solutions of TFA were prepared at four concentrations, e.g., 23, 27, 32, 37.6 g kg^–1^ for isopropanol, 24, 28, 34, 38 g kg^–1^ for ethanol, 13, 15, 18, 21 g kg^–1^ for methanol, 0.8, 1.0, 1.2, 1.5 g kg^–1^ for toluene, and 3, 4, 5.2, 6.2 g kg^–1^ for acetonitrile. TFA was weighted into 20 mL vials using a balance (±0.1 mg accuracy) with the solvents being added to the vials by the mass. The solutions were heated to 50 °C on a stirrer hot plate and held for 30 min under constant agitation to give a homogeneous solution, which was then transferred to 1.5 mL vials using the preheated pipettes.

Polythermal crystallization experiments were carried out using the Technobis Crystal 16 platform,^21,22^ where crystallization and dissolution points were detected by optical turbidity. The solutions were heated and cooled in a preset cycle between −20 to 50 °C with a 1-h holding time at both −20 and +50 °C with each cycle being repeated five times. Experiments were carried out using the same temperature profiles for each concentration and solvent using heating and cooling rates of 0.3, 0.5, 1.0, 1.5, and 2.0 °C min^–1^ and a constant stirring rate of 700 rpm.

The crystals produced in the solution were filtered after solution cooling with the final crystalline forms being analyzed by Fourier transform infrared spectroscopy (Thermo Fisher Scientific iS-10). All the spectral data were collected at ambient temperature with a resolution value of 4 cm^–1^, scan time of 64, and wavenumber ranging from 400 to 4000 cm^–1^. Typical spectra of forms I and II highlighted obvious differences in the peak positions (see Figure S1 in Supporting Information), which were used to characterize the polymorphic forms of the filtered crystals post-crystallization. Most of the crystals obtained were found to be form II albeit in a few cases form I was formed but in this work, only the kinetic data resulting in the former were considered.

#### 2.2.2 Viscosity Measurements

Solution viscosities of TFA for the different solvents were measured as a function of concentration and temperature (10 to 50 °C) using Anton Paar Physica MCR301 for the four solution concentrations listed in Section 2.2.1. The diffusion coefficient for TFA in the different solutions were calculated from the viscosity data using Stokes–Einstein eq 1,^43,44^
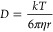
1where *D* is the diffusion coefficient, *T* is the temperature, η is the viscosity of solutions, and *r* is the molecular radius of the solute calculated from the molecular volume based upon the mass of one molecule and the density of the crystal. Note that the radius of the solvated molecule may be a more appropriate parameter for the prediction of the diffusion coefficient. However, it is difficult to give the accurate size of the solvation shell. With the assumption of each solute being solvated with one solvation shell, the radius can be roughly estimated by adding the radius of solute molecule and diameter of solvent molecule. The diffusion coefficients were calculated on the basis of both solute and solvated solute molecular sizes.

#### 2.2.3 Solubility and Crystallizability

**Figure 3:**
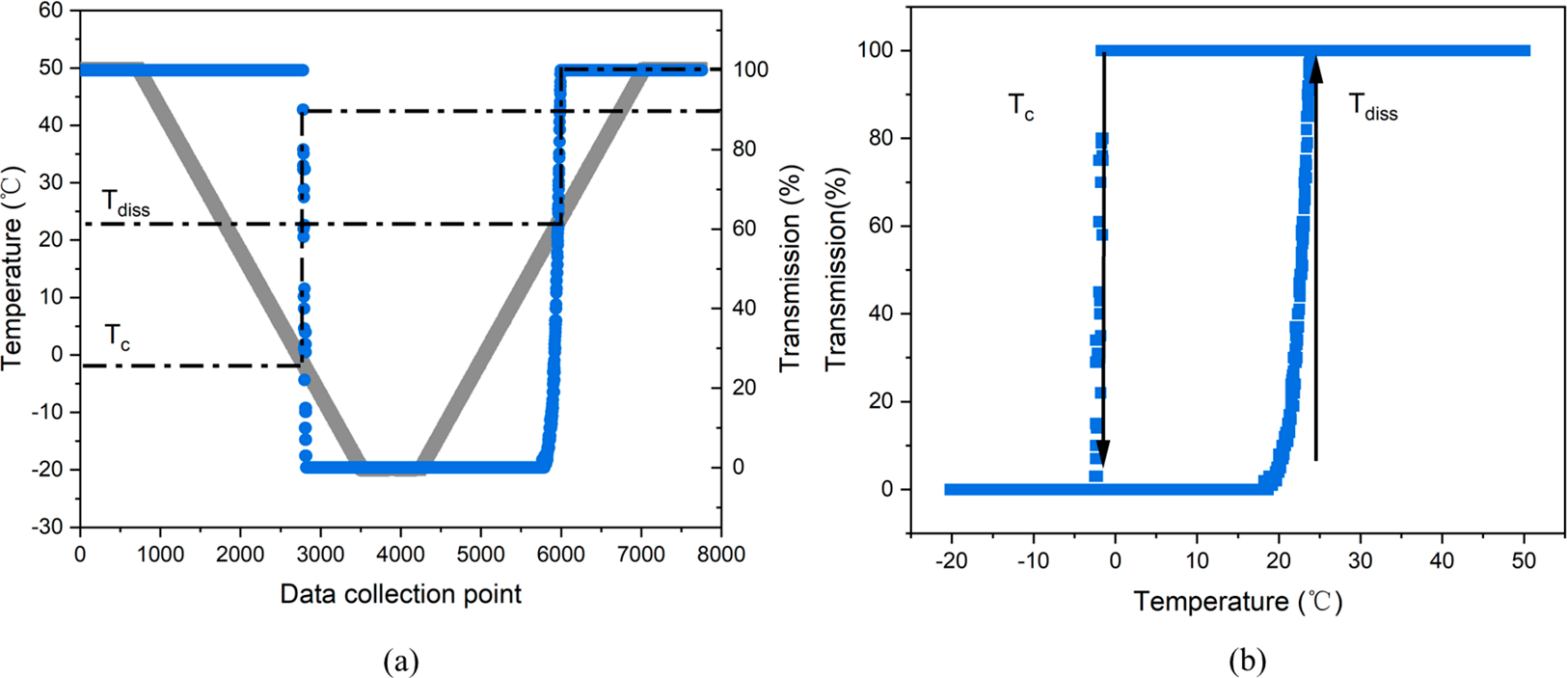
(a) Typical polythermal data from methanol (1.5 × 10^−3^ mol mol^–1^, 0.3 °C min^–1^) for determination of crystallization and dissolution temperatures (*T*_*c*_ and *T*_*diss*_, respectively). (b) Light transmission vs. temperature profile highlighting the extrapolation of *T*_*c*_ and *T*_*diss*_.

Examples of heating–cooling and turbidity profiles are given in Figure 3 (a) and (b), respectively, highlighting the determination of the dissolution (*T*_*diss*_) and crystallization (*T*_*c*_) temperatures. *T*_*c*_ was taken as the onset point where the transmission value dropped below 90% and *T*_*diss*_ was taken as the point where the transmission reached 100%. The equilibrium temperature, *T*_*e*_, was obtained through extrapolating the measured *T*_*diss*_ to 0 °C min^–1^. Then the difference between *T*_*c*_ and *T*_*e*_ was taken as the critical undercooling, Δ*T*_*c*_, which gives the information on crystallizability. The critical supersaturation, *S*_*crit*_, was further calculated using the eq 2, where *x*_*a*_ is the actual molar concentration, and *x*_*c*_ is the equilibrium molar concentration at the critical temperature through extrapolating the *T*_*c*_ to 0 °C min^–1^.
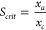
2

Van’t Hoff analysis was performed with the solubility data from the saturation temperatures. The ideal mole solubility (*x*_*ideal*_) was obtained using the Hildebrand equation:^45^
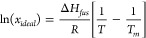
3where *R* is the ideal gas constant, Δ*H*_*fus*_ is the enthalpy of fusion, *T* is the temperature, and *T*_*m*_ is the melting point of the solute. The latter was measured by differential scanning calorimetry through the change of the physical properties (heat flux and temperature of the solute crystals) against time.

The enthalpy (Δ*H*_diss_) and entropy (Δ*S*_diss_) of dissolution were determined based on van’t Hoff equation:
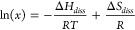
4where *x* is the experimentally measured molar solubility.

The activity coefficients (γ) were estimated from the ratio of the ideal to actual measured solubility data, thus:
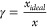
5

#### 2.2.4 Nucleation Kinetics

Polythermal crystallization data was analyzed by the Kashchiev–Borissova–Hammond–Roberts (KBHR) method^26−28^ to determine the nucleation mechanism, i.e. instantaneous or progressive, together with the associated kinetic parameters. In this, the relative critical undercooling, *u*_*c*_, was calculated using eq 6,
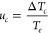
6

The nucleation mechanism was assessed through examination of the nucleation mechanism parameter, ω, which is defined by the slope of ln *u*_*c*_ vs ln *q* from linear regressions,^26−28^ where *q* is the cooling rate. In this, a value of ω < 3 indicates instantaneous nucleation, where all the nuclei appear in the solution at once with crystal growth to follow. A value of ω > 3 signifies progressive nucleation which refers to the continuous formation of new nuclei, where nuclei are formed at different temperatures during crystallization processes; hence, crystal nucleation and growth exist concomitantly.

For the case of progressive nucleation, the final expression of *u*_*c*_(*q*) dependence can be expressed as eq 7:
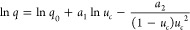
7Plots of ln *q* vs. *u*_*c*_ fitted with eq 7 enables the determination of the nucleation parameters (*q*_0_, *a*_1_ and *a*_2_). The effective interfacial tension (γ_eff_) can then be calculated using *a*_2_ through eq 8:
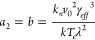
8where *k*_*n*_ is the nuclei shape factor (16π/3 for spherical nuclei), *v*_0_ is the molecular volume in the crystal, and λ is the molecular latent heat of crystallization. The dimensionless thermodynamic parameter related to the nucleation rate, *b*, was also determined^26^ using eq 8.

The critical nucleus radius (r^*^) was determined with the γ_*eff*_ calculated from eq 8, thus:
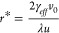
9The nucleation rate constant, *K*_*J*_, can be determined using eq 10 with *q*_0_ fitting parameter determined through eq 7. 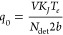
10Here *V* is the volume of the solution, *N*_*det*_ is the number of crystallites formed at the detection point, which can be obtained by performing a mass balance for each solvent system based on the solubility at the crystallization temperature to calculate the total volume of solid nucleated divided by the volume of a single nucleus of the critical radius, r*. Thus the nucleation rate, *J*, was calculated by:
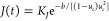
11

### 2.3 Computational Methods: Modeling Solute–Solvent Interactions

Solute–solute and solute–solvent interactions were modeled using intermolecular grid-based search method.^40,41^ See detailed description of the method in Rosbottom et al.^18,42^ This approach operates by considering the interactions between two molecules. One is the substrate located at the center of a spatial grid defined around it, while the second is the probe. Intermolecular interactions are calculated as the probe molecule translates and rotates along the grid. At each molecular position and orientation, the substrate–probe interaction energy was calculated using an experientially derived empirical atom–atom force field approach (Dreiding force field)^46^ together with electrostatic contributions based upon Gasteiger atom point charges,^47,48^ which were also used for cluster energy calculations.

In this study, the conformation in TFA form II structure^49^ were used as the substrate. The solvent molecules (isopropanol, ethanol, methanol, toluene, and acetonitrile) were used as the probe to analyze the solvation properties. Grid optimization was performed and an orthogonal grid shape with sizes 25, 30, and 30 Å in the *X*, *Y* and *Z* axis was found to be suitable for the current study. The number of grid points was set as 10, 15, 15 steps of grid points on the *X*, *Y*, and *Z* axis. The Euler rotational steps were set at 30° for the rotation around the *x*-, *y*- and *z*-axis. Overall, the grid search encompasses a total of 4,866,048 points (location and rotation) for each simulation.

Solvation shell clusters were also built and assessed based upon successive addition of probe molecules whereby the substrate incorporates a probe molecule as located at the lowest energy configuration with the molecular pair becoming the new fixed target for the subsequent probe to find the lowest-energy site, and so on, with the process being repeated until a cluster was built. In this study, a maximum of 10 solvent molecules were used as probes with each solute substrate molecule.

These clusters were optimized using the Forcite Module in BIOVIA Material Studio with the SMART algorithm being used with medium tolerance to distinguish intra- and intermolecular interactions. Normalized cluster energies (*E*_*norm*_) were calculated by scaling respect to the solute/solvent coordination number, *N*_*coor*_, as given in eq 12:
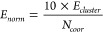
12 In this way, only solvent bound to solute was considered.

## 3 Results and Discussion

### 3.1 Solubility and Solution Thermodynamics

**Figure 4:**
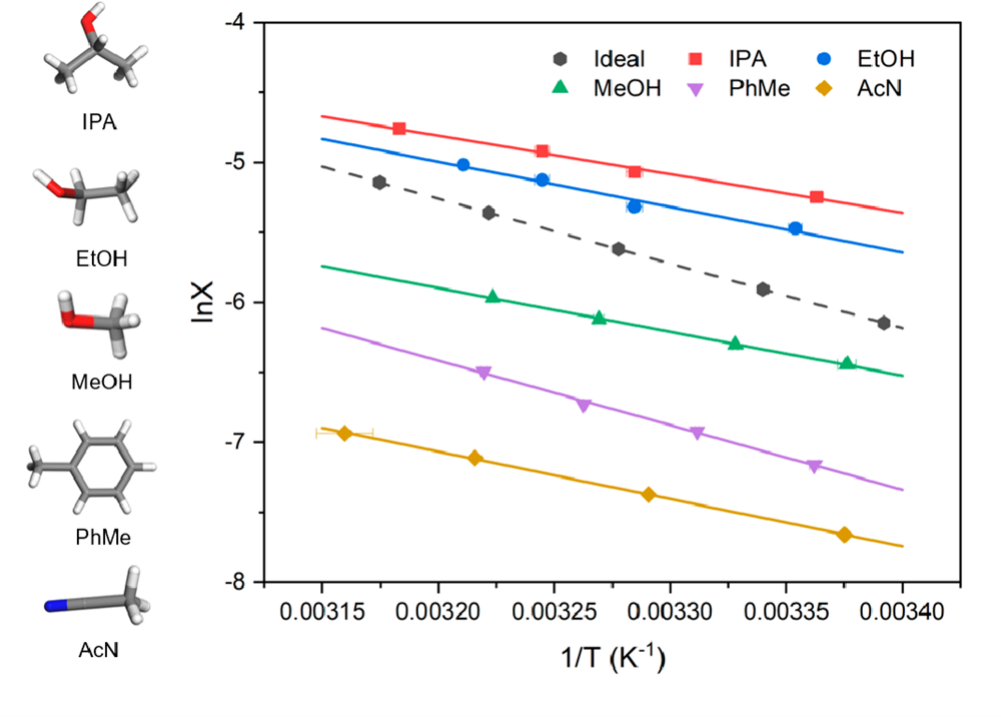
van’t Hoff plots of the solubility in different solvents as obtained from polythermal analysis (solid lines) together with the calculated ideal solubility (dashed line).

The average values for the *T*_*c*_ and *T*_*diss*_ determined as a function of cooling rate *q* and concentration in different solvents are presented in Table S2 (Supporting Information). The solubility data fitted based on the van’t Hoff equation (eq 4) are plotted in Figure 4 with the resultant enthalpy (*ΔH*_*diss*_) and entropy (*ΔS*_*diss*_) of dissolution values and calculated active coefficients (γ) being given in Table 1.

**Table 1:** Calculated Values for the Activity Coefficient γ, Enthalpy of Dissolution Δ*H*_*diss*_, and Entropy of Dissolution Δ*S*_*diss*_ for Different Solvents Based on van’t Hoff Analysis

solvent	γ	Δ*H*_*diss*_ (kJ mol^–1^)	Δ*S*_*diss*_ (kJ K^–1^ mol^–1^)
isopropanol	0.48	22.88	0.033
ethanol	0.62	26.92	0.045
methanol	1.50	26.01	0.034
toluene	3.17	38.36	0.069
acetonitrile	5.03	28.15	0.031

The highest solubility was found in isopropanol with the solute solubilities for the five selected solvents being found to decrease in the following order: isopropanol > ethanol > methanol > toluene > acetonitrile. The data was found to be consistent with the molecular structures of the solvent molecules with the protic solvents isopropanol, ethanol and methanol, acting as both hydrogen bond acceptors and donors, and hence being able to form strong hydrogen-bond interactions with the TFA molecules. A comparison between the 3 alcohols, isopropanol, ethanol, and methanol, suggests that the longer alkyl chain in isopropanol can provide a stronger interaction with the nonpolar phenyl rings in TFA molecule, leading to better solvation of the whole TFA molecules. Thus, the relative solubilities of TFA in the alcohols increases as a function of the alkyl chain length. As an aprotic solvent, acetonitrile exhibits a weaker ability to interact with the polar −COOH group when compared to the protic alcoholic solvents, which is consistent with its lower solubility while toluene, although providing no propensity to form hydrogen bonds, can still solvate the TFA due to the strong inter-rings (π–π) aromatic interactions between the benzene rings in TFA and toluene, consistent with its moderate solubility.

The solubilities in isopropanol and ethanol (Figure 4) were found to be higher than the calculated ideal solubility in the studied temperature range, consistent with the activity coefficient (γ) being less than 1, indicating higher solvent–solute interactions than solvent–solvent and solute–solute interactions. TFA molecules tend to form strong solute/solvent interactions with these two solvents rather than solvent/solvent interactions between the solvent molecules. However, less than ideal behavior was observed for other three solutions in the studied temperature range, where solvent and solute molecules prefer to self-interact rather than mix homogeneously.

### 3.2 Solution Viscosity and Diffusion

**Figure 5:**
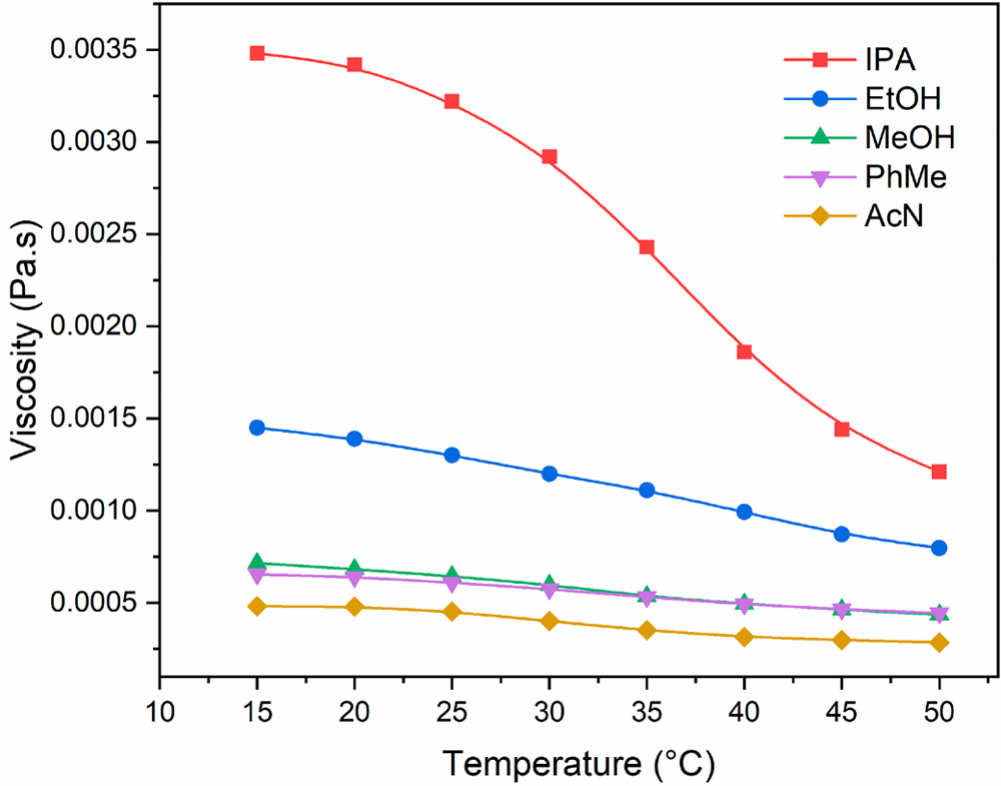
Measured viscosity as a function of temperature in different solvents with a concentration of 23 g kg^–1^ in isopropanol, 24 g kg^–1^ in ethanol, 13 g kg^–1^ in methanol, 2.2 g kg^–1^ in toluene, and 3 g kg^–1^ in acetonitrile. (Selected concentrations are the saturated concentrations at 25 °C for each solvent.)

Figure 5 gives an example of viscosity plots as a function of temperature in different solvents and other viscosity plots with different concentrations can be found in Figure S2 (Supporting Information). In all the solvents, the viscosity was found to decreases with the increase of temperature. Among all the solvents, isopropanol solution was found to show a considerably higher viscosity than other solvents, particularly at lower temperatures, followed by ethanol with a medium viscosity value. Methanol, toluene, and acetonitrile solutions exhibited comparatively lower viscosity with acetonitrile being the lowest. The viscosity was also found to decrease with increasing concentration as expected (Figure S2 (Supporting Information)). However, the effect of solute concentration on viscosity was not found to be as strong an effect as the temperature within the concentration ranges selected, and hence, this concentration effect was not further considered.

Examination of the diffusion coefficient, as calculated using eq 1 based on the solute molecular radius and the viscosity data given in Table 2, reveals the lowest value in isopropanol, suggesting the lowest mass transfer rate of TFA molecules in isopropanol solutions, which might restrict the nucleation process. In contrast, the much lower concentrations in acetonitrile solutions yields the highest diffusion coefficient, i.e., being almost 10 times higher than the value in isopropanol at low temperature. This indicates that TFA molecules might be expected to be quite easy to diffuse within the acetonitrile bulk solutions, making it easier, in turn, to crystallize from this solution, hence indicating higher nucleation rate. Overall, the order of diffusion coefficient is isopropanol < ethanol < methanol ≈ toluene < acetonitrile. This, perhaps not surprisingly, matches the solubility data in a reverse order. The diffusion coefficients estimated based on the solvated molecular radii are also listed in Table S1 (Supporting Information), which have the same trend as shown in Table 2 with the values in Table S1 (Supporting Information) being around 56% smaller. However, a comparison between the diffusion coefficients calculated on the basis of the two methods revealed no significant differences between the overall trends in the data measured as a function of solvent type.

**Table 2:** Diffusion Coefficient of TFA in Different Solutions in the Given Concentration and Temperature (15–50 °C) on the Basis of the TFA Molecular Size

	diffusion coefficient from 15 to 50 °C (10^–10^ m^2^ s^–1^)			
solvent				
isopropanol	23 g kg^–1^	27 g kg^–1^	32 g kg^–1^	37.6 g kg^–1^
	1.46–4.54	1.36–3.87	1.32–4.23	1.08–3.53
ethanol	24 g kg^–1^	28 g kg^–1^	34 g kg^–1^	38 g kg^–1^
	3.28–6.5	3.59–6.9	3.93–6.68	2.55–5.44
methanol	13 g kg^–1^	15 g kg^–1^	18 g kg^–1^	21 g kg^–1^
	7.38–12.88	7.30–12.61	7.17–12.79	6.33–11.86
toluene	0.8 g kg^–1^	1.0 g kg^–1^	1.2 g kg^–1^	1.5 g kg^–1^
	7.39–12.38	7.23–12.38	7.65–11.90	6.72–11.81
acetonitrile	3 g kg^–1^	4 g kg^–1^	5.2 g kg^–1^	6.2 g kg^–1^
	12.08–19.78	10.42–19.29	9.91–19.57	7.23–16.97

### 3.3 Metastable Zone Widths and Crystallizability

**Figure 6:**
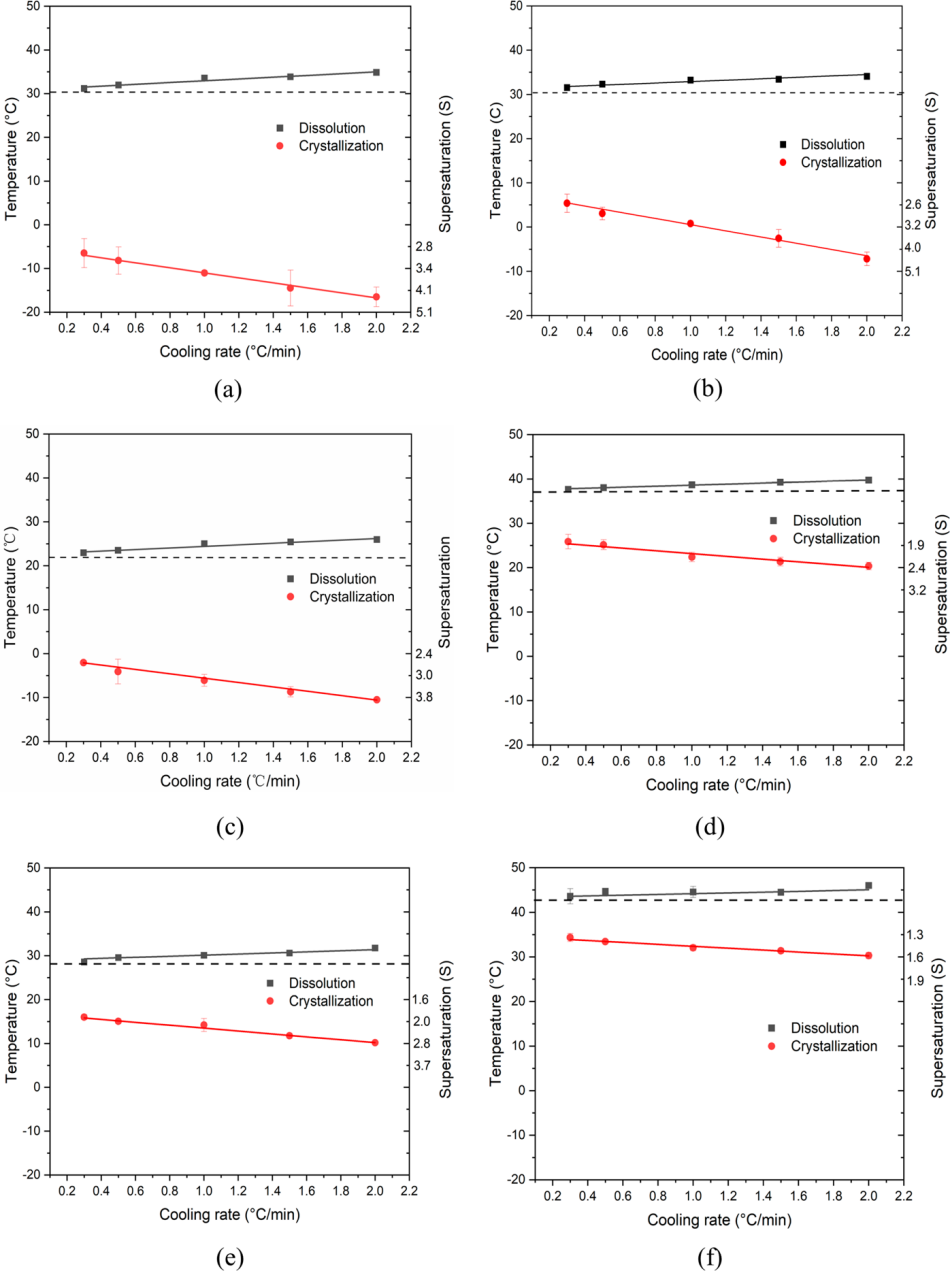
*T*_*diss*_, *T*_*c*_, and calculated supersaturations as a function of cooling rate for mole concentration of (a) 5 × 10^–3^ in isopropanol, (b) 5 × 10^–3^ in ethanol, (c) 1.5 × 10^–3^ in methanol, (d) 1.5 × 10^–3^ in toluene, (e) 1.0 × 10^–3^ in toluene, and (f) 1.0 × 10^–3^ in acetonitrile. The dotted lines indicate the equilibrium temperatures determined by extrapolating the *T*_*diss*_ to 0 °C min^–1^. For comparison purpose, the two TFA concentrations in toluene (d and e) are the same as the concentrations in methanol (c) and in acetonitrile (f), respectively.

Typical plots of *T*_*diss*_ and *T*_*c*_ vs. the cooling rate for solutions in isopropanol, ethanol, methanol, toluene, and acetonitrile are given in Figure 6. See the Supporting Information for a full set of all the plots (Figures S3–S7) together with the results of all the dissolution and crystallization temperatures and calculated Δ*T*_*c*_ for these five solvents (Table S2).

Examination of the data clearly reveals the dependence of the crystallization on-set point on cooling rate with a higher dependence on cooling rate for the alcoholic solutions, consistent with a lower nucleation rate which is rate-limiting with respect to the rate of supersaturation generation. This effect appeared to be less pronounced for solutions with higher solute concentrations suggesting their easier nucleation. In contrast, comparative examination of data measured between the alcohols and the acetonitrile and toluene solutions reveals the latter to exhibit much less variation of crystallization on-set temperature as a function of cooling rate, consistent with TFA nucleating comparatively easier in these solvents.

**Figure 7:**
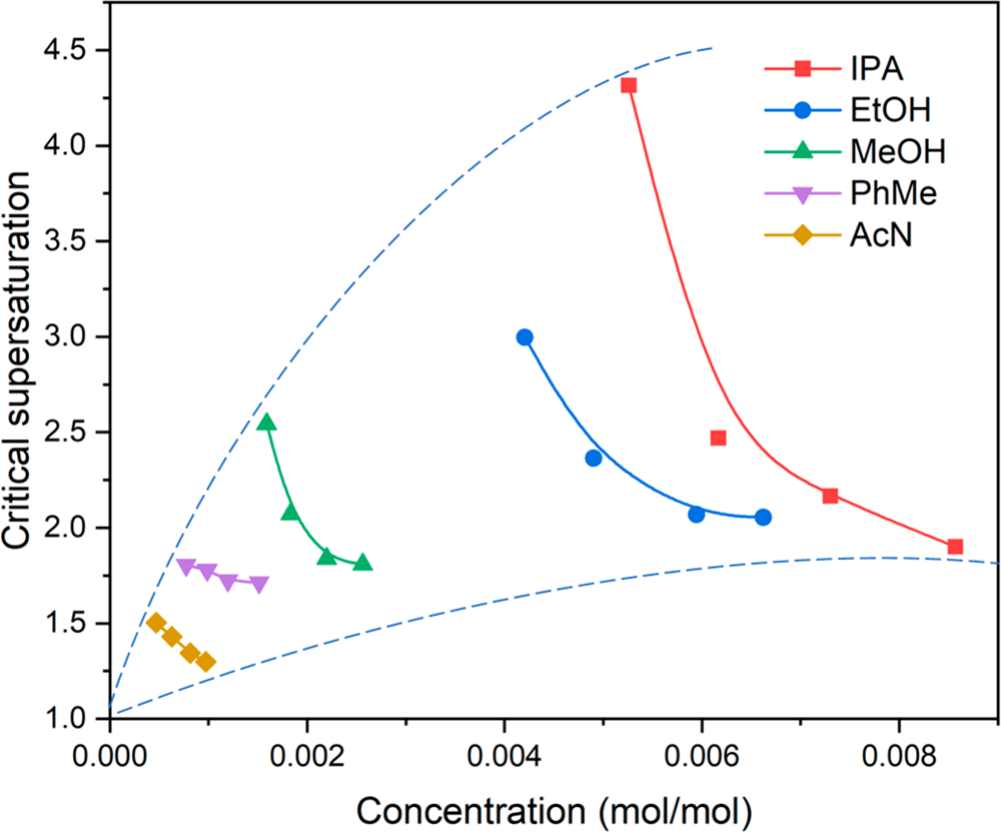
Critical supersaturation of TFA in five solvents as a function of initial solute concentrations, forming a circular sector.

It was also found that TFA had a narrower metastable zone width and lower critical supersaturations (*S*_crit_) for crystallization on-set with the increase of concentration, especially for isopropanol and ethanol solutions. This data is perhaps consistent with the high solute concentration increasing the probability for intermolecular attachment on the surface of nuclei, leading potentially to a higher nucleation rate. Furthermore, the higher concentration is also associated with a higher crystallization temperature where the temperature-dependent viscosity is significantly lower (see section 3.2), increasing the diffusion rate of solute nuclei unit within the bulk solution. Therefore, TFA is comparative easier to nucleate at high concentrations from the perspective of molecular diffusion. This was clearly demonstrated by the data in Figure 7 where, for individual solvents, a higher initial solute concentration, corresponding to higher solution temperature, was found to have lower critical supersaturation and hence lower crystallizability. The more soluble alcoholic solvents (isopropanol, ethanol, and methanol) have big ranges of critical supersaturations with generally higher values compared to other two solvents (toluene and acetonitrile), as shown in the circular sector in Figure 7. Moreover, this data shows the crystallizability of TFA in terms of solvent types. TFA has a wider metastable zone width for crystallization on-set in the polar protic solvents (isopropanol, ethanol, and methanol), consistent with hydrogen bonding between solvent and solute, indicating the difficulty of crystallizing in alcohols, but much less in the polar aprotic acetonitrile and the apolar toluene. The overall difficulty order of nucleation was found to be isopropanol > ethanol > methanol > acetonitrile > toluene, which is consistent with the viscosity measurements in a reverse order, where the high viscosities in the alcoholic solvents limit the molecular diffusion rate and hence solute mass transfer during nucleation, making TFA difficult to nucleate in alcohols.

### 3.4 Nucleation Kinetics and Mechanisms

**Figure 8:**
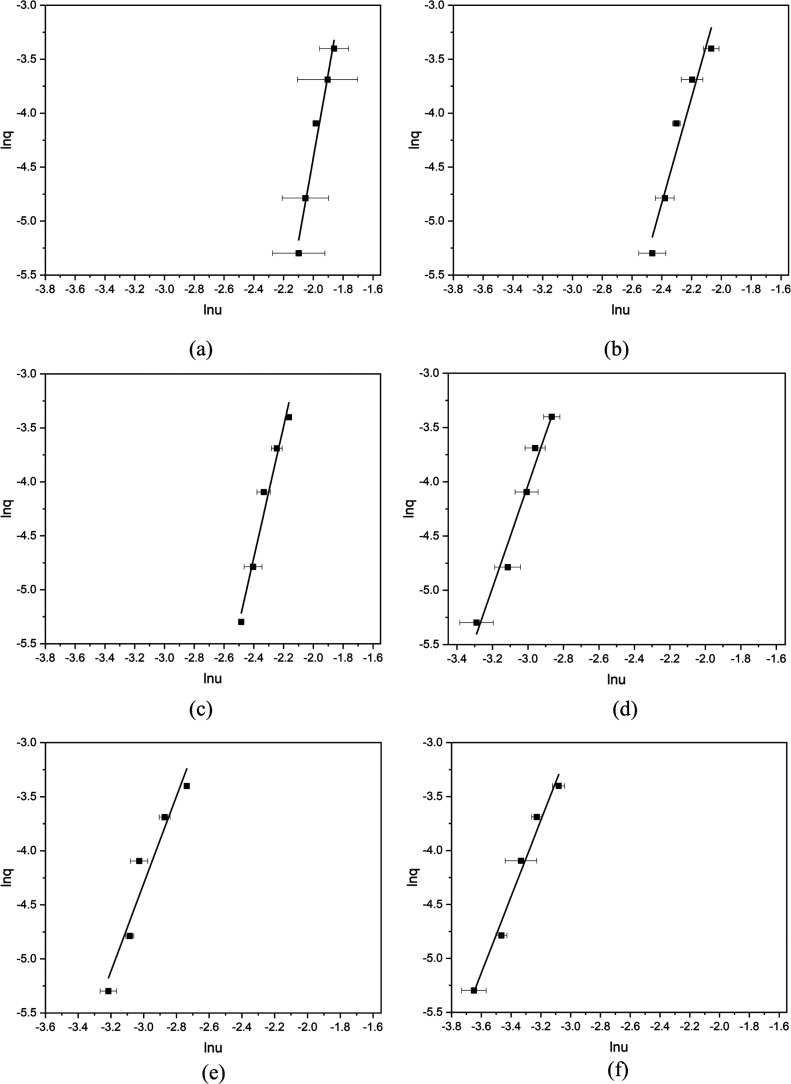
Plot of ln *u* vs. ln *q* for TFA (a) in isopropanol as a mole concentration of 5 × 10^–3^, (b) in ethanol as a mole concentration of 5 × 10^−3^, (c) in methanol as a mole concentration of 1.5 × 10^–3^, (d) in toluene as a mole fraction of 1.5 × 10^–3^ (e) in toluene as a mole concentration of 1.0 × 10^–3^, and (f) in acetonitrile as a mole concentration of 1.0 × 10^–3^. For comparison purpose, the two TFA concentrations in toluene (d and e) are as same as the concentrations in methanol (c) and in acetonitrile (f), respectively.

Representative examples of the nucleation behavior, as assessed with polythermal data analysis, for the different solvents are given in Figure 8 together with a summary of all the results in Table 3. For all the solvents and concentrations studied, the nucleation mechanism parameter (ω) were found to be higher than 3, as would be consistent with a progressive nucleation mechanism. However, analyses of acetonitrile and toluene data were found to be closer to 3, i.e., closer to instantaneous when compared to the alcoholic solvents. Such behavior might reflect the weaker solvent–solute interactions in the former case with the TFA molecules being easier to cluster and self-assemble prior to nucleation as would be consistent with the high activity coefficients of 5.01 and 3.17 (Table 1) for these two solvents, respectively.

**Table 3:** Calculated Nucleation Kinetics of TFA in Isopropanol, Ethanol, Methanol, Toluene, and Acetonitrile Based on the KBHR Analysis^a^

*x*	*T*_e_ (K)	ω	*R*^2^	γ_eff_ (mJ m^–2^)	r* (nm)	range *S*_cryst_	*J* (m^–3^ s ^–1^)
isopropanol							
5.0 × 10^–3^	304.06	7.79	0.96	7.78	0.95–1.21	4.38–4.61	2.86 × 10^8^
6.0 × 10^–3^	307.40	7.82	0.87	6.50	0.89–1.15	2.57–3.69	1.85 × 10^8^
7.0 × 10^–3^	311.20	9.08	0.85	7.69	1.30–1.61	2.24–2.82	1.27 × 10^8^
8.6 × 10^–3^	316.60	8.72	0.98	5.87	1.17–1.45	1.94–2.35	1.83 × 10^9^
ethanol							
4.0 × 10^–3^	297.43	4.54	0.93	4.66	0.55–0.85	3.03–5.91	7.50 × 10^8^
5.0 × 10^–3^	301.97	4.92	0.93	5.73	0.73–1.09	2.55–4.43	2.33 × 10^9^
6.0 × 10^–3^	306.50	5.09	0.98	4.27	0.71–1.02	2.17–3.15	2.77 × 10^9^
6.6 × 10^–3^	310.60	8.22	0.99	5.74	1.00–1.25	2.09–2.62	9.74 × 10^9^
methanol							
1.5 × 10^–3^	295.76	6.08	0.96	5.30	0.77–1.06	2.67–4.02	7.38 × 10^9^
1.8 × 10^–3^	300.47	4.31	0.98	3.73	0.57–0.88	2.21–3.60	3.28 × 10^10^
2.2 × 10^–3^	305.87	4.89	0.98	3.00	0.56–0.84	1.90–2.70	1.60 × 10^10^
2.6 × 10^–3^	310.23	5.11	0.99	3.26	0.70–0.97	1.83–2.34	6.14 × 10^10^
toluene							
0.8 × 10^–3^	297.43	3.80	0.96	3.11	0.57–0.96	1.91–2.90	4.77 × 10^10^
1.0 × 10^–3^	301.97	4.04	0.93	3.93	0.69–1.11	1.80–2.78	3.23 × 10^10^
1.2 × 10^–3^	306.5	3.79	0.96	3.80	0.68–1.10	1.81–2.73	5.88 × 10^10^
1.5 × 10^–3^	310.6	4.73	0.96	2.83	0.56–0.86	1.79–2.58	6.39 × 10^10^
acetonitrile							
0.5 × 10^–3^	296.28	4.94	0.91	2.73	0.77–1.11	1.57–1.93	1.08 × 10^13^
0.6 × 10^–3^	303.89	4.18	0.99	1.81	0.53–0.84	1.47–1.89	2.77 × 10^13^
0.8 × 10^–3^	310.96	3.38	0.99	1.68	0.52–0.93	1.38–1.79	1.25 × 10^13^
1.0 × 10^–3^	316.48	3.54	0.97	1.64	0.55–0.97	1.32–1.67	2.48 × 10^13^

a The nucleation mechanism is progressive for all solvents.

The effective interfacial tensions *(γ*_*eff*_), critical nuclei radius (r*), and the nucleation rate are summarized in Table 3 with the calculated nucleation rates being found to be relatively higher when compared to other organic compounds (see Table S3 (Supporting Information)). A comparison of the nucleation rates between the solvents reveals lower values for the isopropanol and ethanol solvents even for a high level of supersaturation, an observation that further supports the lower crystallizability in these alcoholic solvents. The higher nucleation rates, consistent with the ease of nucleation, as observed in acetonitrile and toluene solvents, correlate well with the lower interfacial tensions observed for these systems.

**Figure 9:**
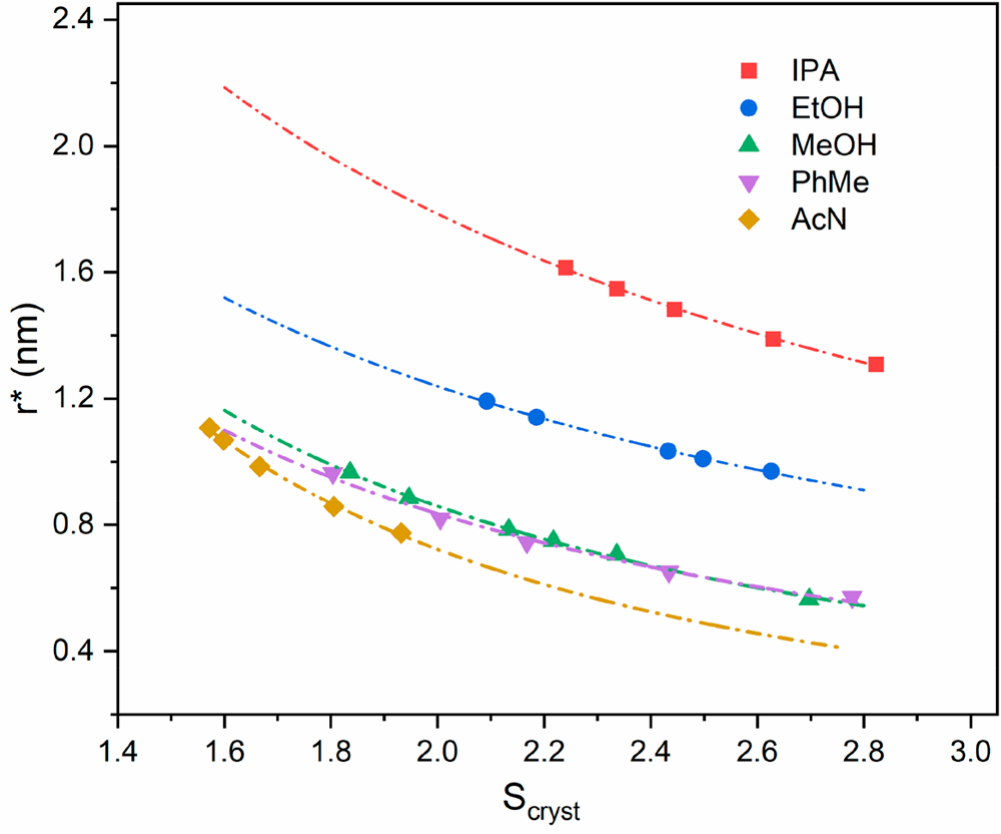
Tendency of critical radius (r^*^) as a function of supersaturation for TFA in isopropanol, ethanol, methanol, toluene, and acetonitrile with the dotted lines for visual propose only.

Examination of the calculated radii for the critical nucleus reveals a negative correlation with the increasing supersaturation for all solvents as expected. Calculation of the cluster sizes between the solvents at the same supersaturation, as shown in Figure 9, reveals the critical sizes to be largest in isopropanol followed by ethanol, methanol, toluene, and acetonitrile, although the relative order of methanol and toluene changed at higher supersaturation levels, i.e., when S was higher than 2.5. The inter-relationship between the critical nucleus size and the interfacial tension was consistent with the interfacial tensions being the rate-limiting step notably in isopropanol with a range of 5.87–7.78 for all supersaturations. Conversely, the interfacial tensions in acetonitrile were found to be relatively low, ranging from 1.64–2.73, which is in agreement with other reported organic molecules as shown in Table S3 (Supporting Information). Overall, the calculated interfacial tensions closely mirror the order of experimentally measured solubility and crystallizability with respect to the solvent types.

With a higher energy being required for solute desolvation and a concomitantly higher energy barrier during nucleation for isopropanol, in contrast, it is acetonitrile where solute desolvation is much easier with the associated energy barrier being lower.

### 3.5 Intermolecular Solute/Solvent Binding Energies

**Figure 10:**
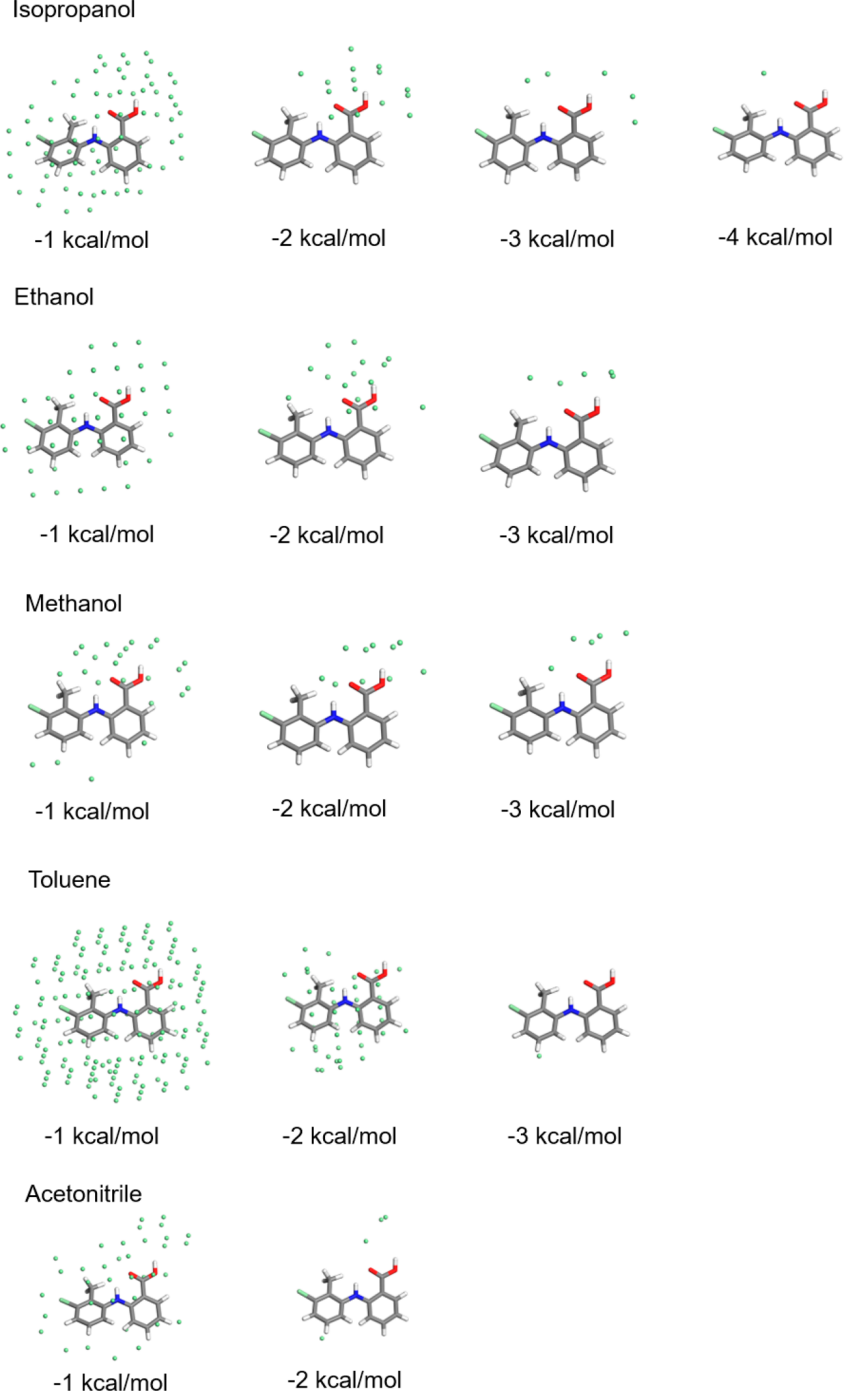
Intermolecular grid-search results showing the strongest solvation sites for five different solvents using different energy filters from −1 to −4 kcal mol^–1^ to highlight and locate likely binding sites. The green spheres represent interaction locations which pass the energy filter.

The results of the solute/solvent binding energies for the five solvents are summarized in Figure 10. The data highlights the binding energy diversity for the different solvents through the use of different low-pass energy filters in assessing the strength of the solvent–solute interactions. These provide an intuitive view regarding the most-favored solvent-binding sites, i.e., identifying which sites can be expected to easily desolvate on energy grounds. For all the solvents except toluene, the strongest interactions were found to be associated with hydrogen bonding to the carboxyl acid group with the intermolecular solute/solvent interactions for the aprotic acetonitrile being weaker than for the protic alcoholic solvents. The interaction energy data for the alcohols show stronger binding to the carboxyl group with the longer chain isopropanol and ethanol being able to solvate nearly all the surface area of the solute. The latter is mainly through weak van der Waals interactions, albeit all the solvation sites were found to be concentrated on the −COOH group for binding energies cutoff of over −2 kcal mol^–1^. In comparison, methanol was not found to completely solvate the whole of the solute molecule, with almost all the binding sites involving binding to carboxylic acid group. In the case of toluene, the strongest binding site was found to be associated with π–π interactions between benzene rings, providing adequate solvation of the whole TFA molecules albeit only through van der Waals interactions.

### 3.6 Solvation Cluster Structures and their Cohesive Energies

**Figure 11:**
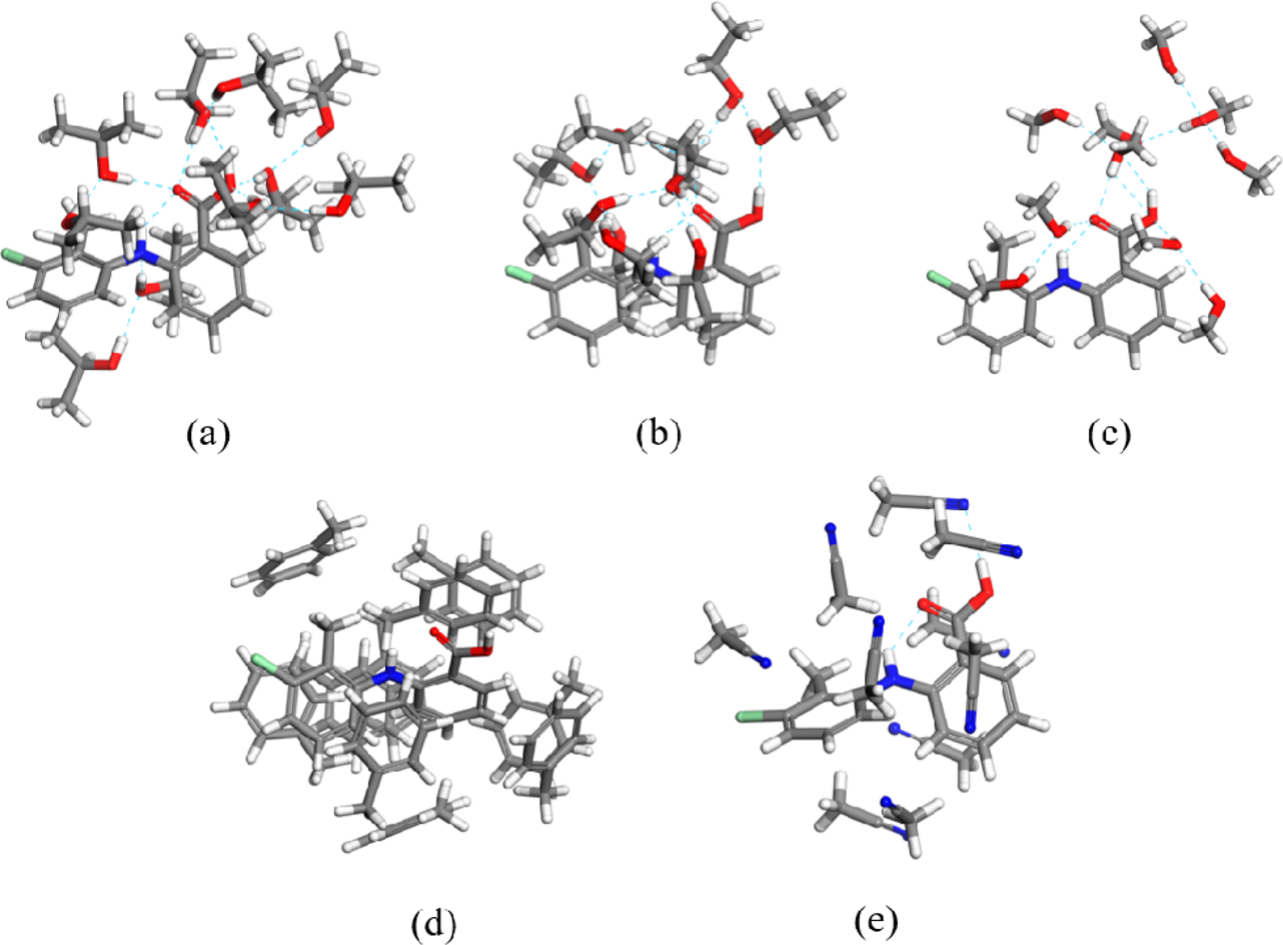
Optimized 10-molecule solvation clusters for (a) isopropanol, (b) ethanol, (c) methanol, (d) toluene, and (e) acetonitrile.

The results for the optimized solvation shell structures are given in Figure 11. The solvation shell structures of isopropanol, ethanol, and methanol form a more anisotropic distribution around TFA molecules with most of the solvent molecules being located around the −COOH group. Comparing the behavior of the three alcohol solvents, methanol was found to exhibit the most anisotropic solvation since the fairly hydrophobic benzene rings of TFA were found to be nearly not solvated at all. With the increase of the alkyl chain length, the isopropanol and ethanol alcohols exhibited a better solvation of all the parts of the TFA molecule mainly due to the increase of van der Waals interactions. Compared to the three alcohols, acetonitrile and toluene were found to show a much more isotropic distribution around the TFA solute molecule, solvating the whole molecule rather than only the polar group.

**Figure 12:**
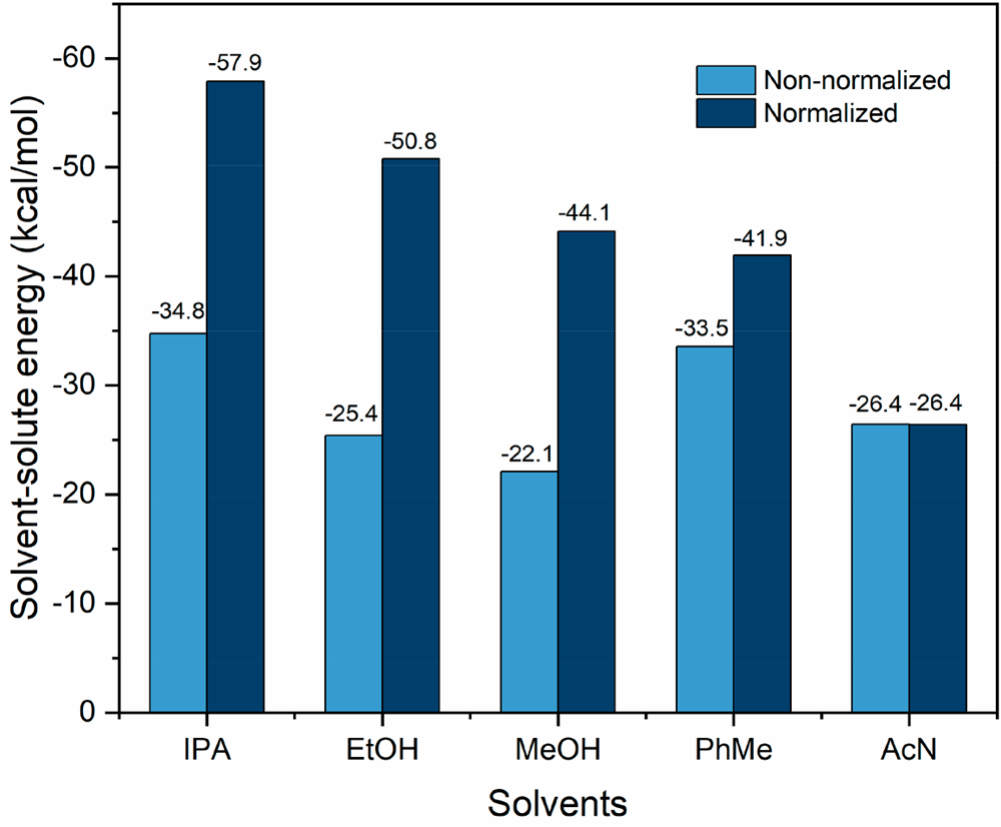
Calculated solvent–solute interaction energies for the 10-molecule solvation clusters of TFA and the normalized interaction energies indicating only solute/solvent interactions, i.e., exclusive solvent molecule self-association.

The calculated solute–solvent interaction energies for the optimized solvation clusters of these five solvents are shown in Figure 12 with further details in Table S4 (Supporting Information). It was found that the alcohol isopropanol has the strongest solute–solvent energies with an energy of −34.8 kcal mol^–1^ followed by toluene with an energy of −33.5 kcal mol^–1^, while methanol exhibits the weakest interactions. It is rational that isopropanol and toluene can have good solvation of the whole solute molecules while methanol only solvates the small carboxyl group for the 10-molecule cluster. Solvents such as acetonitrile, which solvate the whole TFA molecule well, also show higher solvation energies. Actually, these calculated cluster energies do not reflect the real strength of the solute–solvent interaction energies since some of the constituent solvent molecules were found to self-associate and hence did not participate in solute–solvent interactions. To provide a much rational comparison of the strength of solvation interactions, the total solute–solvent energies were also normalized with respect to the solute/solvent coordination numbers. Examination of the normalized data revealed the solvation energies to follow the order: isopropanol > ethanol > methanol > toluene > acetonitrile, closely matching the order of the solubility, where higher solvation energy is related to the higher solubility, and also being consistent with solution diffusivity, effective interfacial tension and crystallizability.

These intermolecular modeling data provide a helpful insight into the solvation state within solution, as well as providing a potential insight into the nucleation behavior at the molecular scale in different solvents. The strength of solute–solvent interactions indicates the ease of desolvation, which is an important factor when considering the nucleation rate of a solute. Strong solute–solvent interactions make it difficult to desolvate during the nucleation process and disrupt the diffusion of TFA molecules into the prenucleation clusters, resulting in a concomitantly slow nucleation rate. Therefore, the strong solvation propensity of isopropanol would be consistent with its difficulty of nucleation. Conversely, the weak solute–solvent interactions in acetonitrile makes it easy for the mass transfer of TFA molecules to the nuclei surface, leading in turn to the ease of nucleation.

### 3.7 Discussion: Interrelating the Data

A comparison between the solubility and nucleation kinetic parameters together with some summarizing remarks are given in Table 4. Summary data is also given in Figure 7, derived from Table 3, which show the critical supersaturation values of TFA in the five solvents as a function of initial solute concentrations.

**Table 4:** Summary Data of TFA in Five Solvents Highlighting Connections between Solution Properties, Nucleation, and Solvation Energies

		isopropanol	ethanol	methanol	toluene	acetonitrile
solution properties	solubility	highest	higher	intermediate	lower	lowest
	diffusion coefficient (10^–10^ m^2^ s^–1^)	1.08–4.54	2.55–6.5	6.33–12.88	6.72–12.38	7.23–19.78
nucleation	ease of nucleation	most difficult	difficult	intermediate	easier	easiest
	MSZW (°C)	23.81–35.95	19.57–26.06	17.09–23.54	10.12–11.54	7.70–10.23
	range of critical supersaturation (*S*_*crit*_)	1.90–4.32	2.05–2.99	1.81–2.54	1.71–1.81	1.29–1.50
	nucleation rate	2.86 × 10^8^ to 1.83 × 10^9^	7.50 × 10^8^ to 9.74 × 10^9^	7.38 × 10^9^ to 6.14 × 10^10^	4.77 × 10^10^ to 6.39 × 10^10^	1.08 × 10^13^ to 2.48 × 10^13^
	γ_eff_ (mJ/m^2^)	5.87–7.78	4.27–5.74	3.00–5.30	2.80–3.93	1.64–2.73
solvation energies	ease of desolvation	most difficult	difficult	intermediate	intermediate	easiest
	energies (kcal/mol)	–57.9	–50.8	–44.1	–41.9	–26.4
comments	The solubility and nucleation are solvent-dependent and highly correlated with each other which can be interpreted by the strength of solvent–solute interactions, where a stronger solvation interaction will lead to a higher solubility and also the difficulty of desolvation during nucleation, hence inhibiting nucleation rate. Furthermore, higher solubility is associated with higher viscosity which limits the molecular diffusion and in turn increases the difficulty of nucleation.					

The measured solubilities of TFA in the five solvents (Table 4, Figure 4) reveal a decreasing order: isopropanol > ethanol > methanol > toluene > acetonitrile, consistent with the calculated active coefficient (γ) in Table 1, supported by the alkyl chain length of the alcohols interacting strongly with nonpolar phenyl rings of TFA molecule, while the π–π interactions between the benzene aromatic rings of toluene and TFA molecules leading to a moderate solubility. Furthermore, the measured diffusivities of TFA in the solvents reveal the lowest diffusion coefficient in isopropanol (Table 2), indicating that it is most difficult for TFA molecules to diffuse within isopropanol solution, and hence also difficult to crystallize, while the reverse case happens for the highest diffusion coefficient in acetonitrile. This matches well with the order of the corresponding active coefficients but in reverse order for solubility data.

Examination of crystallizability and nucleation data reveals that the decreasing order of MSZWs (Table 4) reflecting the same trends as for solubility and effective interfacial tension of crystal nucleus (γ_eff_) data but in reverse order for diffusivity. This is consistent with the existence of hydrogen bonding between TFA and polar protic alcoholic solvents (isopropanol, ethanol, methanol), corresponding to the same order of difficulty level to nucleate which are supported by the same order of nucleation mechanism parameter (ω) as listed in Table 3. Hence all these parameters (solubility, activity coefficient, diffusivity, MSZW, effective interfacial tension, nucleation mechanism parameter) cross-link consistently to result in a reverse order of calculated nucleation rate (Tables 3 and 4), i.e. isopropanol < ethanol < methanol < toluene < acetonitrile.

As shown in Figure 7, higher solubility at the same saturation temperature, hence higher TFA solute concentration, for the five solvents generally requires higher critical supersaturation for nucleation, resulting from the increasing viscosity (decreasing diffusivity) to impede solute mass transfer, hence lowering crystallizability. The highest and lowest TFA concentration ranges in isopropanol and in acetonitrile (Figure 7), respectively, are consistent with their highest and lowest solubilities, corresponding also to their critical supersaturations which exhibit the same trend of the order for solubility. This directly correlates, in turn, to the measured crystallizability and hence nucleation rate, with an order of isopropanol < ethanol < methanol < toluene < acetonitrile, overall, providing a useful diagram to guide the selection of solvent.

The solvation energies (Table 4) calculated for the five solvents demonstrated the difficulty level of desolvation in the order of isopropanol > ethanol > methanol > toluene > acetonitrile, indicating that the intermolecular interactions between TFA and isopropanol molecules are the strongest with solvent acetonitrile being the weakest, hence leading to the former having the highest solubility, effective interfacial tension and nucleation mechanism parameter (Table 3), and widest MSZW, but lowest activity coefficient (Table 1) and nucleation rate (Table 3), with the latter being vice versa.

In summary, the order of the solvation energy closely matches with the solubility (concentration), metastable zone width, effective interfacial tension and nucleation mechanism parameter in the same order, and also aligning well with the activity coefficient, diffusivity, ease of nucleation, and nucleation rate in a reverse order.

## 4 Conclusions

The paper highlights the importance of solvent properties in directing the nucleation kinetics and ease of nucleation in the crystallization process, through influencing the molecular diffusion in bulk solution and the associated desolvation process on the nuclei surface. MSZWs in various protic, aprotic, and apolar solvents were determined, revealing comparatively wide MSZWs when compared to other organic systems. This system was found to nucleate through a progressive mechanism and solvent-dependent behavior with its ease of nucleation being in the following order: isopropanol < ethanol < methanol < toluene < acetonitrile.

The observed solvent-dependent effects upon the crystallizability of TFA were found to be consistent with respect to both a mass transfer and solution chemistry perspective. Analysis of the diffusion coefficients reveals TFA to have the lowest diffusion coefficient in isopropanol, which is nearly 10 times lower than that in acetonitrile, which overall is broadly consistent with the measured nucleation rates, demonstrating important role played by molecular diffusion within the bulk solution in mediating the nucleation of TFA from solutions.

Complementary solvation state predictions using intermolecular modeling reveal the calculated solvation energies and solvation structures to provide a molecular scale insight regarding the solvent-dependent solubility and the solvation strength. This is associated with strong solvation in alcohols, which provides a large energy barrier during the nucleation process, resulting in the difficulty of desolvation in the nucleation process. Conversely, the weaker solvation strength observed in acetonitrile was found to provide an easier pathway for the TFA molecules to adsorb and integrate into the nuclei surface, leading in turn to the ease of nucleation.

Overall, this study has cross-correlated, in detail, solution crystallizability behavior with solution cooling rate, solute concentration and solvent type, combining these with an understanding of the molecular-scale interactions that underpin solvation within the solution phase and, through this, probing the effect of desolvation upon nucleation kinetics. The importance of understanding the structural nature of the solution state at the molecular level in terms of its role in directing the nucleation kinetics of crystallization process has also been highlighted. The work presented here may also provide added-value in crystallization process development through its application in providing an effective triaged-based workflow for use in solvent screening which could be applied for a wider range of organic materials. In this, in-silico solute–solvent interaction modeling together with experimental studies using a restricted range of the cross-linked parameters identified here could be used. Based upon this, a smaller number of potential solvents could be identified and subsequently screened using a wider range of parameters. This approach could, in principle, thus provide an efficient and more sustainable workflow for delivering the solvent selection process in a fast and reliable way.

## List of Symbols

*a*_1_, *a*_2_: KBHR PN parameters
*b*: dimensionless thermodynamic parameter
*C*_*e*_: equilibrium solution concentration (m^–3^)
*D*: diffusion coefficient (m^2^ s^–1^)
*E*_*clustr*_: cluster energy
*E*_*norm*_: normalized cluster energy
Δ*H*_*fus*_: enthalpy of fusion (kJ mol^–1^)
Δ*H*_*diss*_: enthalpy of dissolution (kJ mol^–1^)
*J*(*t*): nucleation rate (m^–3^ s^–1^)
*k*: Boltzmann constant (J K^–1^)
*K*_*J*_: nucleation rate constant (m^–3^ s^–1^)
*k*_*n*_: nucleus numerical shape factor
*N*_*coor*_: coordinate number
*N*_*det*_: detectable number of crystallites
*q*: cooling rate (K s^–1^)
*q*_0_: parameter in the *u*_c_(*q*) dependence for PN (K s^–1^)
*r*: molecular radius (m)
*r*^***^: critical nucleus radius (m)
*R*: ideal gas constant
*R*^2^: coefficient of determination
S: supersaturation
S_crit_: critical supersaturation calculated through extrapolating the *T*_*c*_ to 0 °C min^–1^
S_cryst_: supersaturation for the crystallization onset
Δ*S*_*diss*_: entropy of dissolution (kJ K^–1^ mol^–1^)
*T*: temperature (K)
*T*_*c*_: crystallization temperature (K)
*T*_*diss*_: dissolution temperature (K)
*T*_*e*_: equilibrium dissolution temperature (K)
*T*_*m*_: melting point (K)
Δ*T*_*c*_: critical undercooling for crystallization (K)
*u*: relative undercooling
*u*_*c*_: relative critical undercooling for crystallization
*v*_0_: volume of solute molecule in crystal (m^3^)
*x*: molar fraction
*x*_*a*_: actual molar fraction used for crystallization experiment
*x*_*c*_: equilibrium molar fraction at the extrapolated temperature to 0 °C min^–1^
*x*_*ideal*_: ideal molar solubility
γ: activity coefficient
γ_*eff*_: effective interfacial tension of crystal nucleus (mJ m^–3^)
λ: molecular latent heat of crystallization (J)
η: viscosity
ω: nucleation mechanism parameter, the gradient of the linear dependence of ln *q* with respect to ln *u*_*c*_
AcN: acetonitrile
EtOH: ethanol
IPA: isopropanol
KBHR: Kashchiev–Borissova–Hammond–Roberts
MeOH: methanol
MSZW: metastable zone width
PhMe: toluene
PN: progressive nucleation
TFA: tolfenamic acid

## References

[ref1] Myerson A. S.; Decker S. E.; Fan W. P. Solvent Selection abd Batch Crystallization. Industrial & Engineering Chemistry Process Design and Development 1986, 25 (4), 925–929. 10.1021/i200035a015.

[ref2] Anuar N.; Yusop S. N.; Roberts K. J. Crystallisation of Organic Materials from Solution: A Molecular, Synthonic and Crystallographic Perspective. Crystallography Reviews 2022, 28, 97–215. 10.1080/0889311X.2022.2123916.

[ref3] Davey R. J.; Schroeder S. L. M.; ter Horst J. H. Nucleation of Organic CrystalsA Molecular Perspective. Angew. Chem., Int. Ed. 2013, 52 (8), 2166–2179. 10.1002/anie.201204824. 23307268

[ref4] Sosso G. C.; Chen J.; Cox S. J.; Fitzner M.; Pedevilla P.; Zen A.; Michaelides A. Crystal Nucleation in Liquids: Open Questions and Future Challenges in Molecular Dynamics Simulations. Chem. Rev. 2016, 116 (12), 7078–116. 10.1021/acs.chemrev.5b00744. 27228560 PMC4919765

[ref5] Camacho D. M.; Roberts K. J.; More I.; Lewtas K. Solubility and Nucleation of Methyl Stearate as a Function of Crystallization Environment. Energy Fuels 2018, 32 (3), 3447–3459. 10.1021/acs.energyfuels.7b03212.

[ref6] Turner T. D.; Corzo D. M.; Toroz D.; Curtis A.; Dos Santos M. M.; Hammond R. B.; Lai X.; Roberts K. J. The influence of solution environment on the nucleation kinetics and crystallisability of para-aminobenzoic acid. Phys. Chem. Chem. Phys. 2016, 18 (39), 27507–27520. 10.1039/C6CP04320H. 27711471

[ref7] Rosbottom I.; Toroz D.; Hammond R. B.; Roberts K. J. Conformational and structural stability of the single molecule and hydrogen bonded clusters of para aminobenzoic acid in the gas and solution phases. CrystEngComm 2018, 20 (46), 7543–7555. 10.1039/C8CE00908B.

[ref8] Sullivan R. A.; Davey R. J.; Sadiq G.; Dent G.; Back K. R.; ter Horst J. H.; Toroz D.; Hammond R. B. Revealing the Roles of Desolvation and Molecular Self-Assembly in Crystal Nucleation from Solution: Benzoic and p-Aminobenzoic Acids. Cryst. Growth Des. 2014, 14 (5), 2689–2696. 10.1021/cg500441g.

[ref9] Mealey D.; Zeglinski J.; Khamar D.; Rasmuson A. C. Influence of solvent on crystal nucleation of risperidone. Faraday Discuss. 2015, 179, 309–328. 10.1039/C4FD00223G. 25886651

[ref10] Khamar D.; Zeglinski J.; Mealey D.; Rasmuson A. C. Investigating the Role of Solvent-Solute Interaction in Crystal Nucleation of Salicylic Acid from Organic Solvents. J. Am. Chem. Soc. 2014, 136 (33), 11664–11673. 10.1021/ja503131w. 25029039

[ref11] Muller F. L.; Fielding M.; Black S. A Practical Approach for Using Solubility to Design Cooling Crystallisations. Org. Process Res. Dev. 2009, 13 (6), 1315–1321. 10.1021/op9001438.

[ref12] Liu Y.; Xu S. J.; Zhang X.; Tang W. W.; Gong J. B. Unveiling the Critical Roles of Aromatic Interactions in the Crystal Nucleation Pathway of Flufenamic Acid. Cryst. Growth Des. 2019, 19 (12), 7175–7184. 10.1021/acs.cgd.9b01040.

[ref13] Cruz-Cabeza A. J.; Davey R. J.; Sachithananthan S. S.; Smith R.; Tang S. K.; Vetter T.; Xiao Y. Aromatic stacking - a key step in nucleation. Chem. Commun. 2017, 53 (56), 7905–7908. 10.1039/C7CC02423A. 28660260

[ref14] Zeglinski J.; Kuhs M.; Khamar D.; Hegarty A. C.; Devi R. K.; Rasmuson A. C. Crystal Nucleation of Tolbutamide in Solution: Relationship to Solvent, Solute Conformation, and Solution Structure. Chem. Eur. J. 2018, 24 (19), 4916–4926. 10.1002/chem.201705954. 29431236

[ref15] Toroz D.; Rosbottom I.; Turner T. D.; Corzo D. M. C.; Hammond R. B.; Lai X.; Roberts K. J. Towards an understanding of the nucleation of alpha-para amino benzoic acid from ethanolic solutions: a multi-scale approach. Faraday Discuss. 2015, 179, 79–114. 10.1039/C4FD00275J. 25920721

[ref16] Davey R. J.; Back K. R.; Sullivan R. A. Crystal nucleation from solutions - transition states, rate determining steps and complexity. Faraday Discuss. 2015, 179, 9–26. 10.1039/C5FD00037H. 26022938

[ref17] Jiang S. F.; ter Horst J. H. Crystal Nucleation Rates from Probability Distributions of Induction Times. Cryst. Growth Des. 2011, 11 (1), 256–261. 10.1021/cg101213q.

[ref18] Kaskiewicz P. L.; Rosbottom I.; Camacho Corzo D. M.; Hammond R. B.; Downie R.; Dowding P. J.; George N.; Roberts K. J. Influence of solution chemistry on the solubility, crystallisability and nucleation behaviour of eicosane in toluene: acetone mixed-solvents. CrystEngComm 2021, 23 (17), 3109–3125. 10.1039/D1CE00322D.

[ref19] Kulkarni S. A.; Kadam S. S.; Meekes H.; Stankiewicz A. I.; ter Horst J. H. Crystal Nucleation Kinetics from Induction Times and Metastable Zone Widths. Cryst. Growth Des. 2013, 13 (6), 2435–2440. 10.1021/cg400139t.

[ref20] Rizvi A. K.; Roberts K. J.; Izumi T. The Influence of Supersaturation and the Presence of Biuret on the Nucleation, Growth and Morphology of Urea Crystallised from Ethanolic Solutions. Isr. J. Chem. 2021, 61 (11–12), 727–742. 10.1002/ijch.202100089.

[ref21] Gerson A. R.; Roberts K. J.; Sherwood J. N. An Instrument for the Examination of Nucleation from Solution and its Application to the Study of Precipitation from Diesel Fuels and Solutions of N-alkanes. Powder Technol. 1991, 65 (1–3), 243–249. 10.1016/0032-5910(91)80187-N.

[ref22] Meenan P.; Roberts K. J. The Application of an Automated Crystallization Cell Used to Study the Nucleation Kinetics of Potash Alun. Journal of Materials Science Letters 1993, 12 (22), 1741–1744. 10.1007/BF00517597.

[ref23] Taggart A. M.; Voogt F.; Clydesdale G.; Roberts K. J. An examination of the nucleation kinetics of n-alkanes in the homologous series C13H28 to C32H66, and their relationship to structural type, associated with crystallization from stagnant melts. Langmuir 1996, 12 (23), 5722–5728. 10.1021/la9600816.

[ref24] Smith L. A.; Roberts K. J.; Machin D.; McLeod G. An examination of the solution phase and nucleation properties of sodium, potassium and rubidium dodecyl sulphates. J. Cryst. Growth 2001, 226 (1), 158–167. 10.1016/S0022-0248(01)01368-9.

[ref25] Tang X.; Kaskiewicz P. L.; Camacho Corzo D. M.; Lai X.; Roberts K. J.; Dowding P.; More I. Solubility and crystallisability of the ternary system: Hexadecane and octadecane representative in fuel solvents. Fuel 2018, 226, 665–674. 10.1016/j.fuel.2018.04.022. PMC6141768 30246070

[ref26] Kashchiev D.; Borissova A.; Hammond R. B.; Roberts K. J. Effect of cooling rate on the critical undercooling for crystallization. J. Cryst. Growth 2010, 312 (5), 698–704. 10.1016/j.jcrysgro.2009.12.031. 20369862

[ref27] Kashchiev D.; Borissova A.; Hammond R. B.; Roberts K. J. Dependence of the Critical Undercooling for Crystallization on the Cooling Rate. J. Phys. Chem. B 2010, 114 (16), 5441–5446. 10.1021/jp100202m. 20369862

[ref28] Camacho Corzo D. M. C.; Borissova A.; Hammond R. B.; Kashchiev D.; Roberts K. J.; Lewtas K.; More I. Nucleation mechanism and kinetics from the analysis of polythermal crystallisation data: methyl stearate from kerosene solutions. CrystEngComm 2014, 16 (6), 974–991. 10.1039/C3CE41098F.

[ref29] Lopez-Mejias V.; Kampf J. W.; Matzger A. J. Polymer-Induced Heteronucleation of Tolfenamic Acid: Structural Investigation of a Pentamorph. J. Am. Chem. Soc. 2009, 131 (13), 4554–4555. 10.1021/ja806289a. 19334766 PMC2729806

[ref30] Sacchi P.; Reutzel-Edens S. M.; Cruz-Cabeza A. J. The unexpected discovery of the ninth polymorph of tolfenamic acid. CrystEngComm 2021, 23 (20), 3636–3647. 10.1039/D1CE00343G.

[ref31] Case D. H.; Srirambhatla V. K.; Guo R.; Watson R. E.; Price L. S.; Polyzois H.; Cockcroft J. K.; Florence A. J.; Tocher D. A.; Price S. L. Successful Computationally Directed Templating of Metastable Pharmaceutical Polymorphs. Cryst. Growth Des. 2018, 18 (9), 5322–5331. 10.1021/acs.cgd.8b00765.

[ref32] Mattei A.; Li T. Interplay between molecular conformation and intermolecular interactions in conformational polymorphism: a molecular perspective from electronic calculations of tolfenamic acid. Int. J. Pharm. 2011, 418 (2), 179–86. 10.1016/j.ijpharm.2011.04.062. 21570454

[ref33] Mattei A.; Li T. Polymorph formation and nucleation mechanism of tolfenamic acid in solution: an investigation of pre-nucleation solute association. Pharm. Res. 2012, 29 (2), 460–70. 10.1007/s11095-011-0574-7. 21879384

[ref34] Mattei A.; Mei X.; Miller A.-F.; Li T. Two Major Pre-Nucleation Species that are Conformationally Distinct and in Equilibrium of Self-Association. Cryst. Growth Des. 2013, 13 (8), 3303–3307. 10.1021/cg401026j.

[ref35] Mattei A.; Li T. Nucleation of Conformational Polymorphs: A Computational Study of Tolfenamic Acid by Explicit Solvation. Cryst. Growth Des. 2014, 14 (6), 2709–2713. 10.1021/cg5000815.

[ref36] Du W.; Cruz-Cabeza A. J.; Woutersen S.; Davey R. J.; Yin Q. Can the study of self-assembly in solution lead to a good model for the nucleation pathway? The case of tolfenamic acid. Chem. Sci. 2015, 6 (6), 3515–3524. 10.1039/C5SC00522A. 29511513 PMC5814770

[ref37] Tang W.; Mo H.; Zhang M.; Parkin S.; Gong J.; Wang J.; Li T. Persistent Self-Association of Solute Molecules in Solution. J. Phys. Chem. B 2017, 121 (43), 10118–10124. 10.1021/acs.jpcb.7b07763. 29017013

[ref38] Roberts K. J.; Sherwood J. N.; Stewart A. The Nucleation of N-eicosane Crystals from Solution in N-dodecane in the Presence of Homologous Impurities. J. Cryst. Growth 1990, 102, 419–426. 10.1016/0022-0248(90)90400-F.

[ref39] Gerson A. R.; Roberts K. J.; Sherwood J. N.; Taggart A. M.; Jackson G. The Role of Growth Environment on the Crystallisation of Normal alkanes in the Homologous Series from C18H38 to C29H60. J. Cryst. Growth 1993, 128, 1176–1181. 10.1016/S0022-0248(07)80119-9.

[ref40] Hammond R. B.; Ma C. Y.; Roberts K. J.; Ghi P. Y.; Harris R. K. Application of systematic search methods to studies of the structures of urea-dihydroxy benzene cocrystals. J. Phys. Chem. B 2003, 107 (42), 11820–11826. 10.1021/jp035010b.

[ref41] Hammond R. B.; Hashim R. S.; Ma C. Y.; Roberts K. J. Grid-based molecular modeling for pharmaceutical salt screening: Case example of 3,4,6,7,8,9-hexahydro-2H-pyrimido (1,2-a) pyrimidinium acetate. J. Pharm. Sci. 2006, 95 (11), 2361–2372. 10.1002/jps.20657. 16886182

[ref42] Rosbottom I.; Pickering J. H.; Hammond R. B.; Roberts K. J. A Digital Workflow Supporting the Selection of Solvents for Optimizing the Crystallizability of p-Aminobenzoic Acid. Org. Process Res. Dev. 2020, 24 (4), 500–507. 10.1021/acs.oprd.9b00261.

[ref43] Einstein A. The motion of elements suspended in static liquids as claimed in the molecular kinetic theory of heat. Annalen Der Physik 1905, 322 (8), 549–560. 10.1002/andp.19053220806.

[ref44] Leroy F. Molecular Driving Forces. Statistical Thermodynamics in Biology, Chemistry, Physics, and Nanoscience, 2nd edition. Soft Mater. 2013, 11 (2), 231–231. 10.1080/1539445X.2011.619612.

[ref45] Prausnitz J. M., Ed.; Molecular Thermodynamics of Fluid Phase Equilibrium; Prentice Hall Inc.: 1969.

[ref46] Mayo S. L.; Olafson B. D.; Goddard W. A. Dreiding: A Generic Force Field for Molecular Simulations. J. Phys. Chem. 1990, 94, 8897–8909. 10.1021/j100389a010.

[ref47] Gasteiger J.; Marsili M. New Model for Calculating Atomic Charges in Molecules. Tetrahedron Lett. 1978, 19 (34), 3181–3184. 10.1016/S0040-4039(01)94977-9.

[ref48] Gasteiger J.; Marsili M. Iterative Partial Equalization of Orbital Electronegativity-A Rapid Access to Atomic Charge. Tetrahedron 1980, 36 (22), 3219–3228. 10.1016/0040-4020(80)80168-2.

[ref49] Andersen K. V.; Larsen S.; Alhede B.; Gelting N.; Buchardt O. Characterization of Two Polymorphic Forms of Tolfenamic Acid, N-(2-methyl-3-chlorophenyl)anthranilic acid: Their Crystal Structures and Relative Stabilities. Journal of the chemical society, Perkin Transactions 2 1989, 10, 1443–1447. 10.1039/p29890001443.

