# Supplementary material for: The Influence of Solvent Selection upon the Crystallizability and Nucleation Kinetics of Tolfenamic Acid Form II
Source: Cryst Growth Des. 2023 Jul 17;23(8):5846–59. doi: 10.1021/acs.cgd.3c00450 (PMC10401637; doi:10.1021/acs.cgd.3c00450)
Supplement: Supplementary file 1 — cg3c00450_si_001.pdf [file cg3c00450_si_001.pdf]

Supporting Information

The Influence of Solvent Selection upon the Crystallisability and Nucleation Kinetics of Tolfenamic Acid Form II §

Yu Liu,[1, 2] Cai Y. Ma,[2] Junbo Gong,[1] and Kevin J. Roberts[2,*]

[1] State Key Laboratory of Chemical Engineering, Tianjin University, Tianjin, 300072, China.

[2] Centre for the Digital Design of Drug Products, School of Chemical and Process Engineering, University of Leeds, Woodhouse Lane, Leeds LS2 9JT, UK

The supplementary material supports the main manuscript by providing further details of the following: **Figure S1** presents FTIR spectrum for form I, form II and the mixture. **Figure S2** shows Solution viscosity as a function of temperature with different concentrations and different solvents. **Figure S3-S7** shows T_{diss}, T_c and partial supersaturation as a function of cooling rate with different concentrations and solvents. **Table S1** presents Diffusion coefficients of TFA in different solutions in the given concentration and temperature on the basis of the solvated solvent molecular sizes. **Table S2** gives Average T_{diss}, T_c and ΔT_c for TFA in different solvents at different

cooling/heating rate and different concentrations. **Table S3** provides Solute-solvent interaction energies for the 10-molecule solvation clusters of TFA.

FTIR Results

FTIR are used to determine the forms of the product. As shown in Figure S1, the FTIR spectrum for different forms show obvious different in the range of 700 – 800 cm^{-1}. Form I shows peaks at 741 cm^{-1} and 768 cm^{-1}, while form II at 748 cm^{-1} and 777 cm^{-1}. The mixture of form I and form II shows both the peaks of form I and form II, make it easy to determine the polymorphs of the product using FTIR.

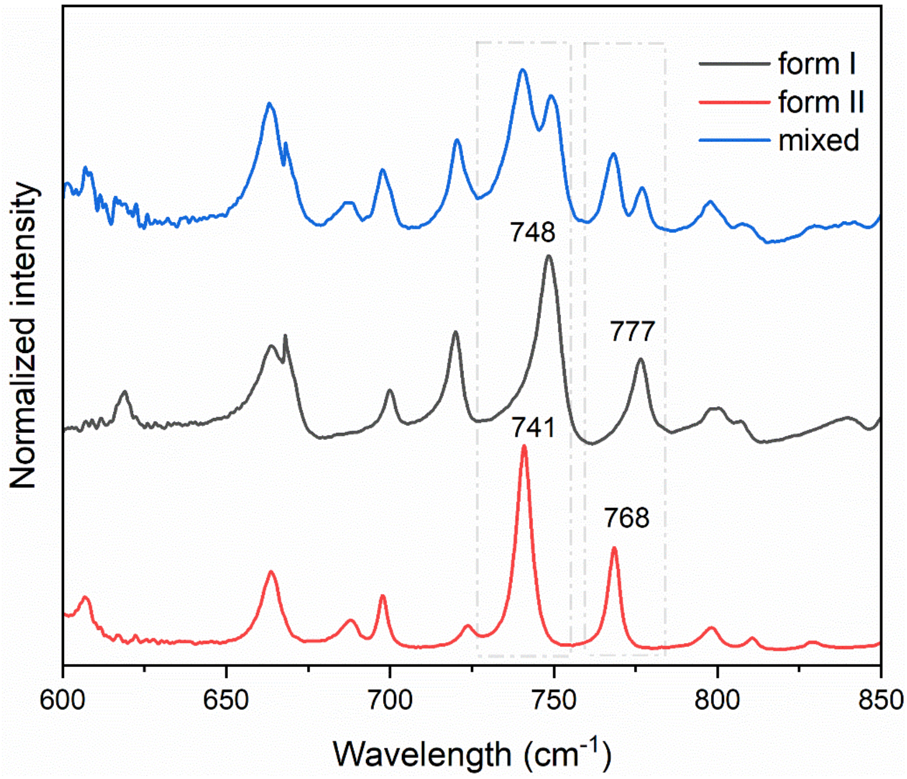

Figure S1. A standard FTIR spectrum for form I, form II and mixture (form I: form II = 1:1) of TFA.

Viscosity Measurements

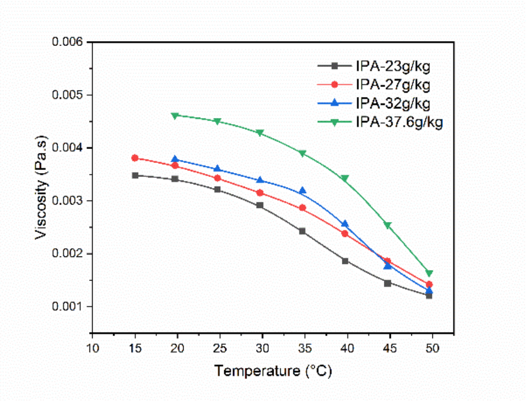

(a)

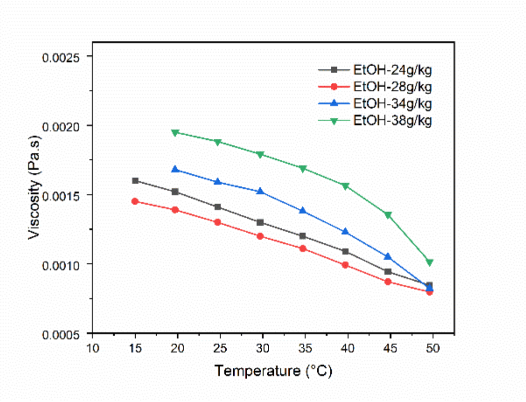

(b)

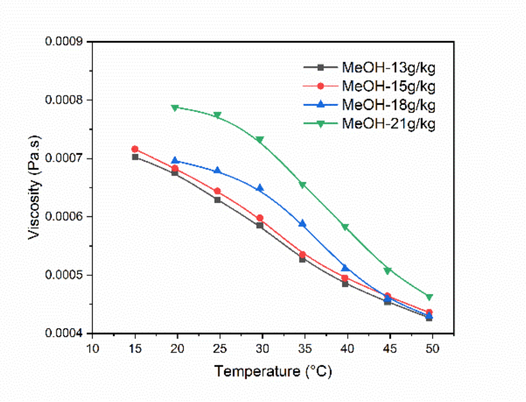

(c)

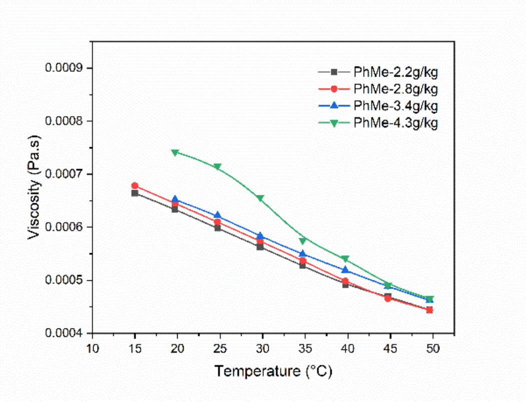

(d)

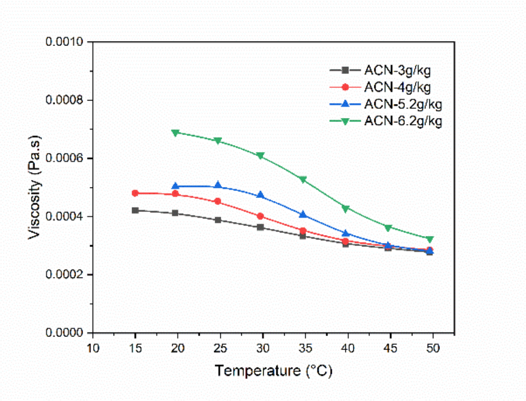

(e)

Figure S2. Solution viscosity as a function of temperature with different concentrations in (a) isopropanol, (b) ethanol, (c) methanol, (d) toluene and (e) acetonitrile.

Table S1. Diffusion coefficients of TFA in different solutions in the given concentration and temperature on the basis of the solvated solvent molecular sizes.

Solvent	Diffusion coefficient from 20 °C to 50 °C (10^{-10} m^2 s^{-1})			
Isopropanol	23 g kg^{-1}<br>0.59~1.86	27 g kg^{-1}<br>0.55~1.58	32 g kg^{-1}<br>0.53~1.73	37.6 g kg^{-1}<br>0.44~1.37
Ethanol	24 g kg^{-1}<br>1.41~2.80	28 g kg^{-1}<br>1.54~2.97	34 g kg^{-1}<br>1.46~2.87	38 g kg^{-1}<br>1.10~2.34
Methanol	13 g kg^{-1}<br>3.39~5.93	15 g kg^{-1}<br>3.36~5.80	18 g kg^{-1}<br>3.30~5.88	21 g kg^{-1}<br>2.91~5.46
Toluene	0.8 g kg^{-1}<br>3.01~4.73	1.0 g kg^{-1}<br>2.95~4.73	1.2 g kg^{-1}<br>2.92~4.54	1.5 g kg^{-1}<br>2.56~4.51
Acetonitrile	3 g kg^{-1}<br>5.30~8.68	4 g kg^{-1}<br>4.57~8.46	5.2 g kg^{-1}<br>4.35~8.58	6.2 g kg^{-1}<br>3.17~7.44

Dissolution and Crystallisation Temperature

Average dissolution (T_{diss}) and crystallisation (T_c) temperatures for TFA in different solvents at each cooling/heating rate (q) and each solution concentration are shown in Table S2. The value of the equilibrium dissolution temperature, T_e, is also provided for each concentration from extrapolation of dissolution temperatures to 0°/min cooling rate. The calculated values critical undercooling, ΔT_c, is also presented in Table 1 for each cooling rate in the specified solvent system. The plots of T_{diss} and T_c as a function of cooling rate in different solvents with each solution concentration were shown in Figures S3 - S6.

Table S2. Average dissolution (T_{diss}), crystallisation temperatures (T_c) and ΔT_c for TFA in different solvents at different cooling/heating rate and different concentration. The standard deviations are basing at least 5 repeats.

**Isopropanol**<br>23.0 g kg^{-1} Rate (°C min^{-1})	T_{diss} (°C)	T_{crys} (°C)	ΔT_c (°C)
0.3	31.18±0.33	-6.48±3.33	37.39
0.5	31.97±0.30	-8.20±3.14	39.11

1	33.58±0.39	-11.02±0.55	41.93
1.5	33.86±0.39	-14.50±4.10	45.41
2	34.86±0.28	-16.50±2.24	47.41
	T_e = 30.91		
27.0 g kg^{-1} Rate (°C min^{-1})	T_{diss} (°C)	T_{crys} (°C)	ΔT_c (°C)
0.3	35.10±0.09	1.20±3.40	33.05
0.5	37.99±0.51	-0.80±3.83	35.05
1	37.85±0.22	-3.20±1.90	37.45
1.5	38.63±0.12	-5.93±1.40	40.18
2	40.31±0.25	-8.30±2.43	42.55
	T_e = 34.25		
32.0 g kg^{-1} Rate (°C min^{-1})	T_{diss} (°C)	T_{crys} (°C)	ΔT_c (°C)
0.3	37.86±0.15	9.88±3.77	28.17
0.5	38.79±0.13	8.66±3.63	29.39
1.0	41.28±0.22	7.36±2.07	30.69
1.5	42.57±0.06	5.29±2.95	32.76
2.0	42.55±0.06	3.31±1.41	34.74
	T_e = 38.05		
37.6 g kg^{-1} Rate (°C min^{-1})	T_{diss} (°C)	T_{crys} (°C)	ΔT_c (°C)
0.3	43.72±1.77	18.96±4.33	24.49
0.5	44.66±0.82	17.86±1.90	25.59
1.0	45.34±2.28	15.94±1.40	27.51
1.5	47.13±0.51	14.08±4.40	29.37
2.0	47.53±0.73	13.21±0.72	30.24
	T_e = 43.45		

**Ethanol**<br>24.0 g kg^{-1} Rate (°C min^{-1})	T_{diss} (°C)	T_{crys} (°C)	ΔT_c (°C)
0.3	24.62±0.05	-2.10±1.29	26.29
0.5	25.43±0.05	-7.0±3.38	31.19
1	27.17±0.21	-11.20±1.87	35.39
1.5	27.57±0.22	-13.20±1.14	37.39

2	27.92±0.14	-16.48±1.10	40.67
	T_e = 24.19		
28.0 g kg^{-1} Rate (°C min^{-1})	T_{diss} (°C)	T_{crys} (°C)	ΔT_c (°C)
0.3	31.54±0.22	5.40±2.06	25.90
0.5	32.32±0.41	3.09±1.41	28.21
1	33.25±0.22	0.82±0.60	30.48
1.5	33.44±0.46	-2.55±1.99	33.85
2	34.12±0.43	-7.19±1.54	38.49
	T_e = 31.30		
34.0 g kg^{-1} Rate (°C min^{-1})	T_{diss} (°C)	T_{crys} (°C)	ΔT_c (°C)
0.3	35.69±2.10	14.21±1.49	20.83
0.5	36.02±0.14	12.56±1.01	22.48
1.0	37.64±0.19	9.93±2.15	25.11
1.5	38.13±0.13	6.90±2.27	28.15
2.0	39.45±0.21	4.98±1.08	30.07
	T_e = 35.04		
38.0 g kg^{-1} Rate (°C min^{-1})	T_{diss} (°C)	T_{crys} (°C)	ΔT_c (°C)
0.3	41.76±3.19	17.98±2.76	23.03
0.5	41.58 ±0.05	16.84±2.15	24.17
1.0	42.53±0.54	14.09±1.7	26.92
1.5	42.93±0.17	13.41±1.86	27.61
2.0	43.19±0.18	12.15±3.00	28.86
	T_e = 41.01		

**Methanol** 13.0 g kg^{-1} Rate (°C min^{-1})	T_{diss} (°C)	T_{crys} (°C)	ΔT_c (°C)
0.3	22.97±0.19	-2.05±0.38	24.66
0.5	23.53±0.12	-4.09±3.82	26.70
1	25.04±0.4	-6.09±1.35	28.70
1.5	25.44±0.16	-8.70±1.16	31.31
2	25.98±0.2	-11.36±0.42	33.97
	T_e = 22.61		

15.0 g kg^{-1} Rate (°C min^{-1})	T_{diss} (°C)	T_{crys} (°C)	ΔT_c (°C)
0.3	27.69±0.14	5.99±3.2	21.34
0.5	28.63±0.26	3.90±0.89	23.42
1	29.82±0.11	0.47±0.48	26.85
1.5	27.69±0.14	-3.14±1.15	30.46
2	29.82±0.11	-5.71±1.4	33.03
	T_e = 27.32		
18.0 g kg^{-1} Rate (°C min^{-1})	T_{diss} (°C)	T_{crys} (°C)	ΔT_c (°C)
0.3	32.98±1.14	14.48±2.42	18.24
0.5	33.26±0.05	12.53±0.27	20.19
1.0	34.43±0.14	9.88±0.47	22.84
1.5	34.72±0.08	7.89±0.75	24.83
2.0	34.90±0.09	5.54±0.79	27.18
	T_e = 32.72		
21.0 g kg^{-1} Rate (°C min^{-1})	T_{diss} (°C)	T_{crys} (°C)	ΔT_c (°C)
0.3	37.26±0.15	19.58±1.50	17.50
0.5	37.57±0.05	17.99±0.64	19.09
1.0	38.26±0.14	15.52±0.19	21.56
1.5	38.84±0.62	14.50±0.28	22.58
2.0	38.92±0.17	13.12±0.23	23.96
	T_e = 37.08		

**Toluene**<br>2.2 g kg^{-1} Rate (°C min^{-1})	T_{diss} (°C)	T_{crys} (°C)	ΔT_c (°C)
0.3	24.69±0.08	13.38±0.87	10.90
0.5	25.08±0.04	11.50±0.76	12.78
1	26.66±0.13	10.14±0.04	14.09
1.5	27.12±0.54	8.14±0.27	16.09
2	27.44±0.09	5.89±1.86	18.39
	T_e = 24.28		
2.8 g kg^{-1} Rate (°C min^{-1})	T_{diss} (°C)	T_{crys} (°C)	ΔT_c (°C)
0.3	28.56±0.50	16.71±0.08	12.11

0.5	29.57±0.08	15.03±0.39	13.79
1	30.09±0.14	14.21±1.47	14.61
1.5	30.61±0.13	11.75±0.58	17.07
2	31.76±0.23	9.28±0.14	19.54
	T_e = 28.82		
3.4 g kg^{-1} Rate (°C min^{-1})	T_{diss} (°C)	T_{crys} (°C)	ΔT_c (°C)
0.3	33.67±0.08	21.27±2.06	12.08
0.5	33.82±0.23	20.19±1.02	13.16
1.0	34.46±0.15	17.40±0.61	14.89
1.5	35.95±0.13	15.64±0.75	17.71
2.0	35.42±0.23	13.72±0.33	19.63
	T_e = 33.35		
4.3 g kg^{-1} Rate (°C min^{-1})	T_{diss} (°C)	T_{crys} (°C)	ΔT_c (°C)
0.3	37.66±0.11	25.87±1.64	11.58
0.5	38.04±0.15	23.64±1.32	13.81
1.0	38.66±0.10	22.09±1.07	15.36
1.5	39.26±0.05	21.34±0.97	16.11
2.0	39.73±0.04	19.75±0.84	17.7
	T_e = 37.45		

**Acetonitrile**<br>3.0 g kg^{-1} Rate (°C min^{-1})	T_{diss} (°C)	T_{crys} (°C)	ΔT_c (°C)
0.3	23.52±0.10	11.82±1.63	11.31
0.5	25.51±0.29	11.42±0.83	11.71
1	26.43±0.08	10.43±0.47	12.70
1.5	26.51±0.76	8.54±0.78	14.59
2	27.00±0.21	6.96±0.89	16.17
	T_e = 23.13		
4.0 g kg^{-1} Rate (°C min^{-1})	$\mathrm{T_{dis}}$ (°C)	$\mathrm{T_{crys}}$ (°C)	$\Delta \mathrm{T_c}$ (°C)
0.3	31.24±1.20	20.56±1.5	10.18
0.5	31.69±0.05	19.24±0.49	11.50
1	33.38±0.21	17.55±0.64	13.19

1.5	33.82±0.32	15.86±0.86	14.88
2	34.55±0.05	14.31±0.05	16.1
	T_e = 30.74		
5.2 g kg^{-1} Rate (°C min^{-1})	T_{diss} (°C)	T_{crys} (°C)	ΔT_c (°C)
0.3	36.68±1.53	29.14±1.14	8.67
0.5	38.35±0.09	27.45±1.11	10.36
1.0	38.89±1.82	25.86±0.91	11.95
1.5	39.61±0.35	24.02±1.03	13.79
2.0	39.94±0.09	22.27±0.5	15.54
	T_e = 37.81		
6.2 g kg^{-1} Rate (°C min^{-1})	T_{diss} (°C)	T_{crys} (°C)	ΔT_c (°C)
0.3	43.60±1.70	35.10±0.89	8.23
0.5	44.69±0.25	33.43±0.35	9.90
1.0	44.56±1.25	32.04±0.64	11.29
1.5	44.50±0.05	30.78±0.49	12.55
2.0	46.02±0.18	28.79±0.69	14.54
	T_e = 43.33		

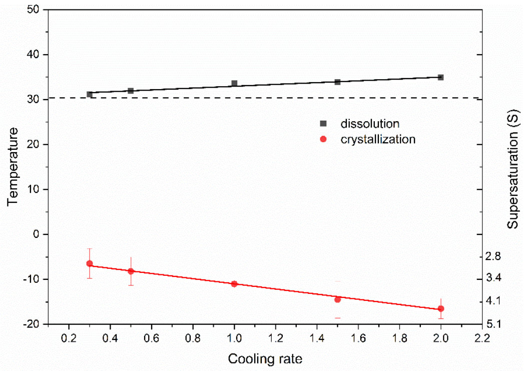

(a)

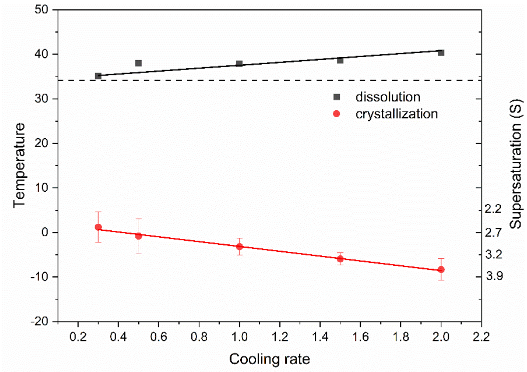

(b)

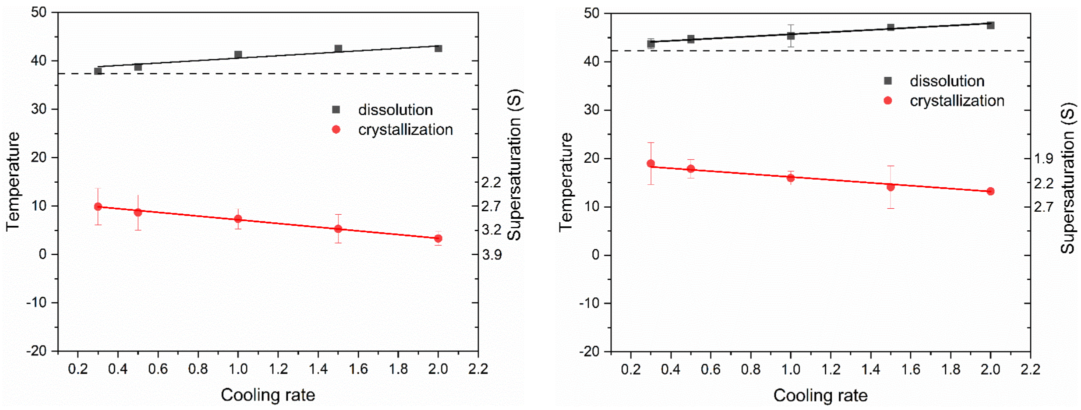

(c) (d)

Figure S5. T_{diss}, T_c and partial supersaturation as a function of cooling rate in isopropanol for mole concentration of (a) 5×10^{-3}, (b) 6×10^{-3}, (c) 7×10^{-3} and (d) 8.6×10^{-3}.

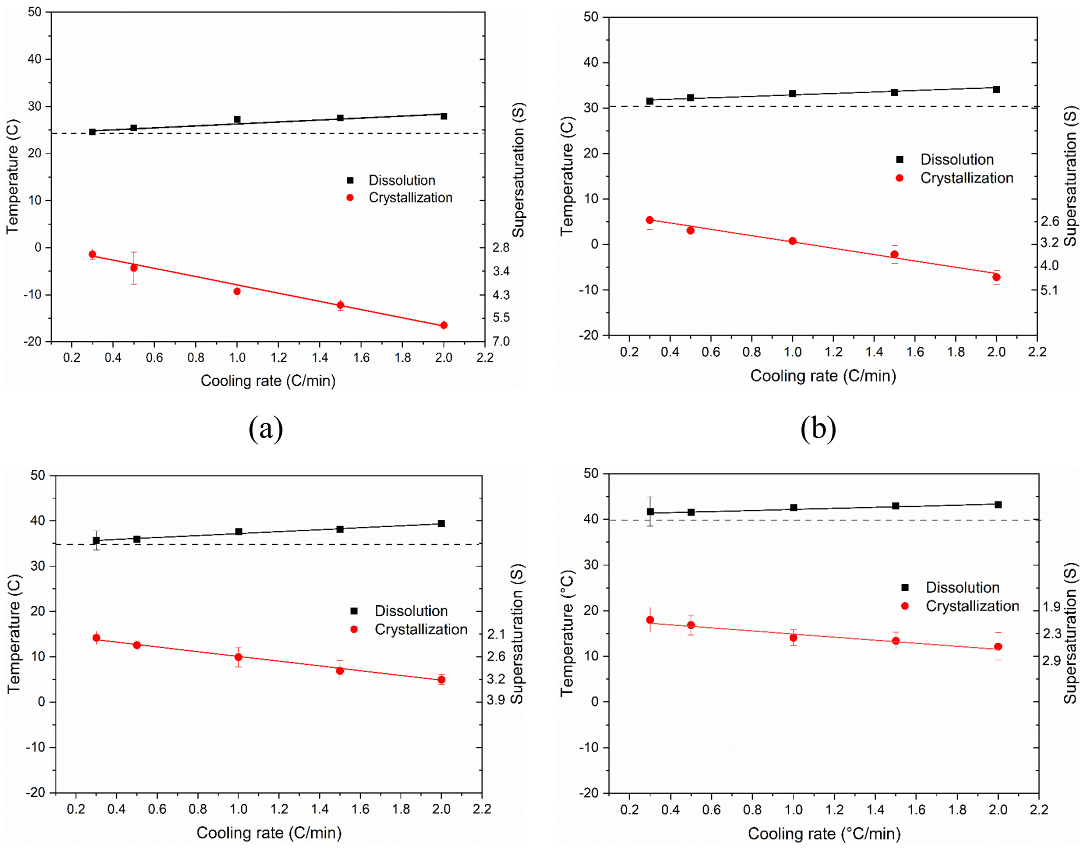

(a) (b)

(c) (d)

Figure S4. T_{diss}, T_c and partial supersaturation as a function of cooling rate in ethanol for mole concentration of (a) 4×10^{-3}, (b) 5×10^{-3}, (c) 6×10^{-3} and (d) 6.6×10^{-3}.

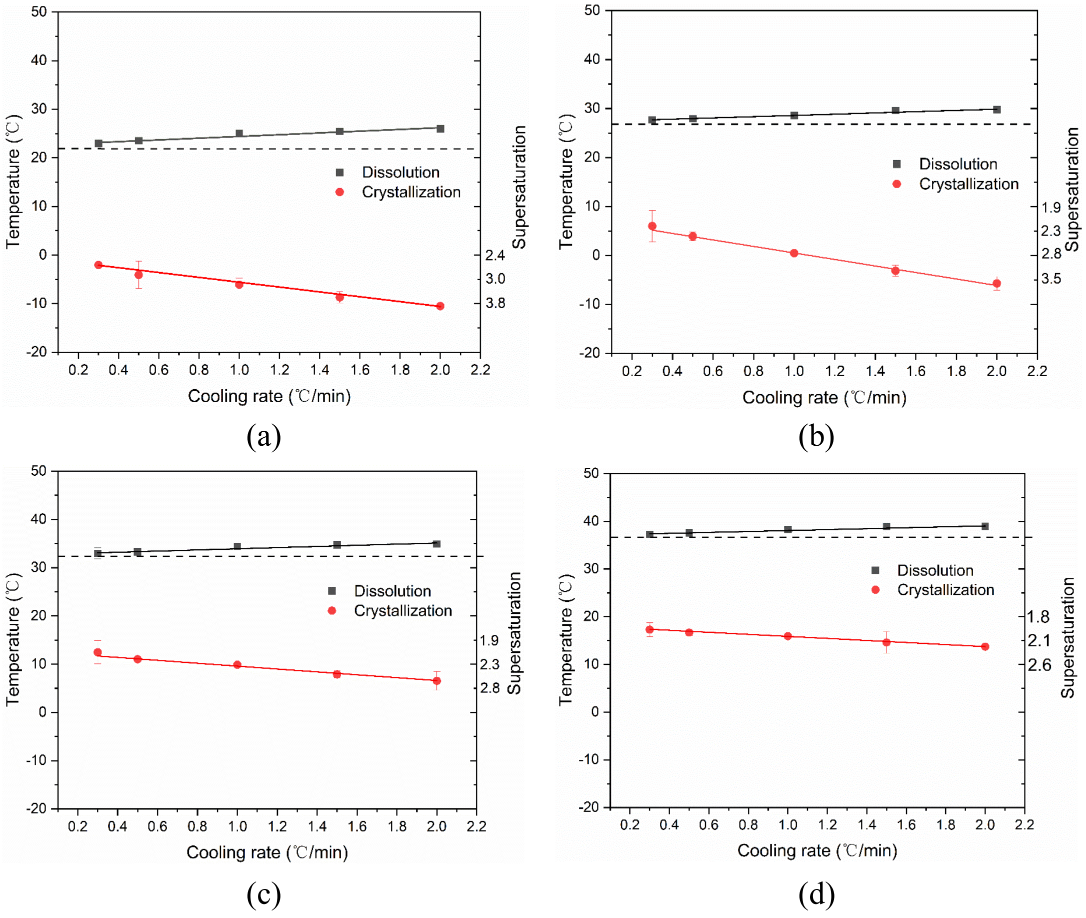

Figure S3. T_{diss}, T_c and partial supersaturation as a function of cooling rate in methanol for mole concentration of (a) 1.5×10^{-3}, (b) 1.8×10^{-3}, (c) 2.2×10^{-3} and (d) 2.6×10^{-3}.

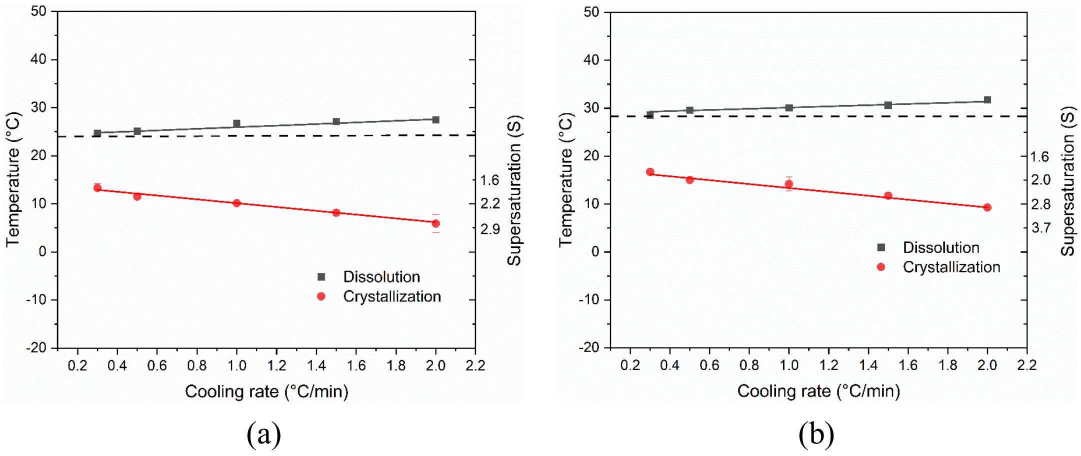

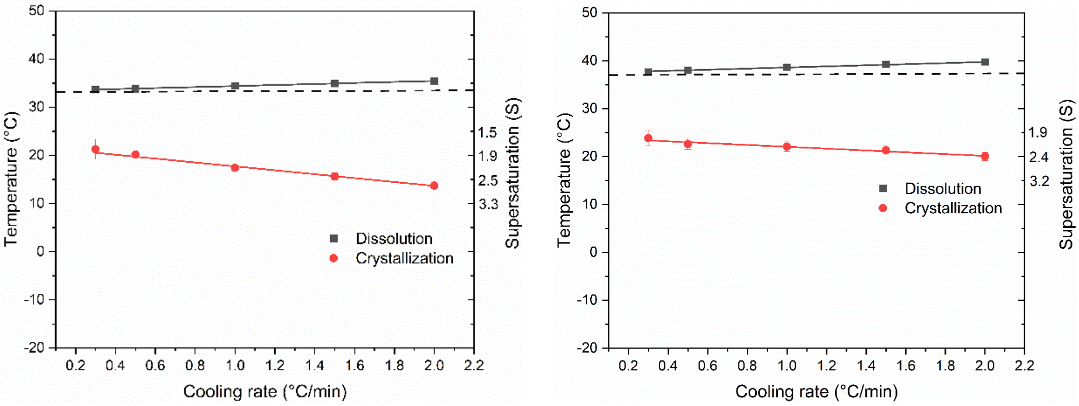

(c) (d)

Figure S7. T_{diss}, T_c and partial supersaturation as a function of cooling rate in toluene for mole concentration of (a) 0.8×10^{-3}, (b) 1.0×10^{-3}, (c) 1.2×10^{-3} and (d) 1.5×10^{-3}.

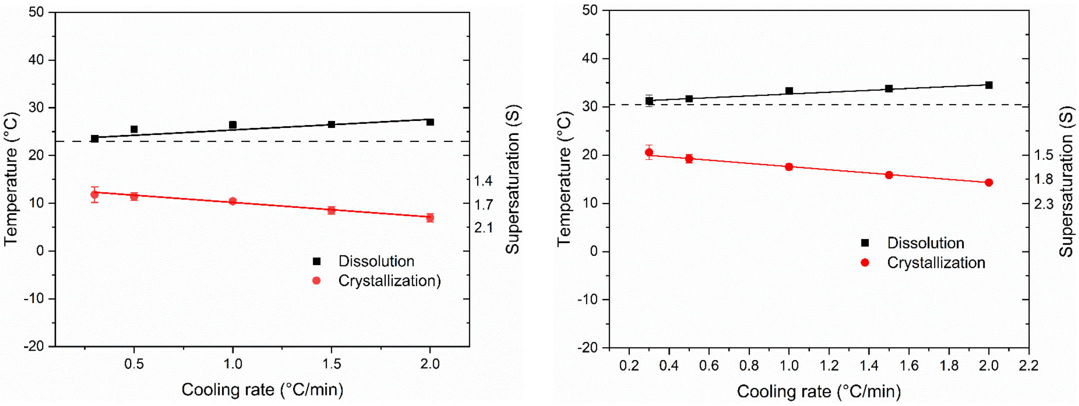

(a) (b)

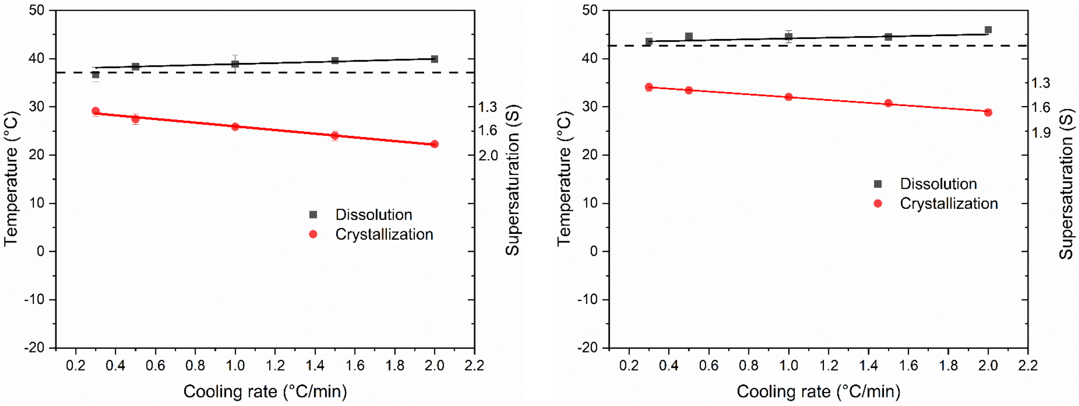

(c) (d)

Figure S6. T_{diss}, T_c and partial supersaturation as a function of cooling rate in acetonitrile for mole concentration of (a) 0.5×10^{-3}, (b) 0.6×10^{-3}, (c) 0.8×10^{-3} and (d) 1.0×10^{-3}.

Table S3. Reported nucleation rates for other organic compounds.

Compounds	Method	J (m^{-3} s^{-1})	γ_{eff} (mJ m^{-2})
Methyl stearate in toluene[1]	Polythermal method	4.5×10^{22} ~ 10.4×10^{22}	1.18~1.7
Para-aminobenzoic acid in ethanol[2]	Polythermal method		1.3~2.7
Benzoic in methanol[3]	Isothermal method	18 - 1698	2.7
m-aminobenzoic acid in ethanol[4]	Isothermal method	50 - 1220	8.7
L-histidine in water[4]	Isothermal method	160 - 1980	5.1
Flufenamic acid in acetonitrile[5]	Isothermal method	1037 - 3060	0.8
H4EDTA in water[6]	Isothermal method	0.23×10^{12} ~ 3.9×10^{12}	21
Isonicotinamide in ethanol[7]	Polythermal method	268	

Intermolecular Grid-Search Modeling

Table S4. Solute-solvent interaction energies for the 10-molecule solvation clusters of TFA.

Solvent	Coordination Number	Solute-Solvent Energy (kcal mol^{-1}) Original	Normalized
Isopropanol	6	-34.8	-57.9
Ethanol	5	-25.4	-50.8
Methanol	5	-22.1	-44.1
Toluene	7	-33.5	-41.9
Acetonitrile	10	-26.4	-26.4

References

(1) Camacho, D. M.; Roberts, K. J.; More, I.; Lewtas, K., Solubility and Nucleation of Methyl Stearate as a Function of Crystallization Environment. *Energy & Fuels* **2018,** 32, (3), 3447-3459.

(2) Turner, T. D.; Corzo, D. M.; Toroz, D.; Curtis, A.; Dos Santos, M. M.; Hammond, R. B.; Lai, X.; Roberts, K. J., The influence of solution environment on the nucleation kinetics and crystallisability of para-aminobenzoic acid. *Phys Chem Chem Phys* **2016,** 18, (39), 27507-27520.

(3) Cruz-Cabeza, A. J.; Davey, R. J.; Sachithananthan, S. S.; Smith, R.; Tang, S. K.; Vetter, T.; Xiao, Y., Aromatic stacking - a key step in nucleation. *Chemical Communications* **2017,** 53, (56), 7905-7908.

(4) Jiang, S. F.; ter Horst, J. H., Crystal Nucleation Rates from Probability Distributions of Induction

Times. *Crystal Growth & Design* **2011,** 11, (1), 256-261.
(5) Liu, Y.; Xu, S. J.; Zhang, X.; Tang, W. W.; Gong, J. B., Unveiling the Critical Roles of Aromatic Interactions in the Crystal Nucleation Pathway of Flufenamic Acid. *Crystal Growth & Design* **2019,** 19, (12), 7175-7184.
(6) Roelands, C. P. M.; Roestenberg, R. R. W.; ter Horst, J. H.; Kramer, H. J. M.; Jansens, P. J., Development of an experimental method to measure nucleation rates in reactive precipitation. *Crystal Growth & Design* **2004,** 4, (5), 921-928.
(7) Kulkarni, S. A.; Kadam, S. S.; Meekes, H.; Stankiewicz, A. I.; ter Horst, J. H., Crystal Nucleation Kinetics from Induction Times and Metastable Zone Widths. *Crystal Growth & Design* **2013,** 13, (6), 2435-2440.
